# Estimation of area- and mass-based leaf nitrogen contents of wheat and rice crops from water-removed spectra using continuous wavelet analysis

**DOI:** 10.1186/s13007-018-0344-1

**Published:** 2018-08-29

**Authors:** Dong Li, Xue Wang, Hengbiao Zheng, Kai Zhou, Xia Yao, Yongchao Tian, Yan Zhu, Weixing Cao, Tao Cheng

**Affiliations:** National Engineering and Technology Center for Information Agriculture (NETCIA), Key Laboratory of Crop System Analysis and Decision Making, Ministry of Agriculture and Rural Affairs, Jiangsu Key Laboratory for Information Agriculture, Jiangsu Collaborative Innovation Center for Modern Crop Production, Nanjing Agricultural University, One Weigang, Nanjing, Jiangsu 210095 China

**Keywords:** Nitrogen content, Water-removed, Wavelet analysis, Shortwave infrared

## Abstract

**Background:**

The visible and near infrared region has been widely used to estimate the leaf nitrogen (N) content based on the correlation of N with chlorophyll and deep absorption valleys of chlorophyll in this region. However, most absorption features related to N are located in the shortwave infrared (SWIR) region and the physical mechanism of leaf N estimation from fresh leaf reflectance spectra remains unclear. The use of SWIR region may help us reveal the underlying mechanism of casual relationships and better understand the spectral responses to N variation from fresh leaf reflectance spectra. This study combined continuous wavelet analysis (CWA) and water removal technique to improve the estimation of N content and leaf mass per area (LMA) by reducing the effect of water absorption and enhancing absorption signals in the SWIR region. The performance of the wavelet-based method was evaluated for estimating leaf N content and LMA of rice and wheat crops from fresh leaf reflectance spectra collected over a 2-year field experiment and compared with normalization difference (ND)-based spectral indices.

**Results:**

The LMA and area-based N content (N_area_) exhibited better correlations with the determined wavelet features derived from the water-removed (WR) spectra (LMA: *R*^2^ = 0.71, N_area_: *R*^2^ = 0.77) than those from the measured reflectance (MR) spectra (LMA: *R*^2^ = 0.62, N_area_: *R*^2^ = 0.64). The wavelet features performed remarkably better than the optimized ND indices for the estimations of LMA and N_area_ with MR spectra or WR spectra. Based on the best estimations of LMA and N_area_ with wavelet features from WR spectra, the mass-based N content (N_mass_) could be retrieved with a high accuracy (*R*^2^ = 0.82, RMSE = 0.32%) in the indirect way. This accuracy was higher than that for N_mass_ obtained in the direct use of a single wavelet feature (*R*^2^ = 0.68, RMSE = 0.42%).

**Conclusions:**

The enhancement of absorption features in the SWIR region through the CWA applied to water-removed (WR) spectra was able to improve the spectroscopic estimation of leaf N content and LMA as compared to that obtained with the reflectance spectra of fresh leaves. The success in estimating LMA and N with this method would advance the spectroscopic estimations of grain quality parameters for staple crops and individual dry matter constituents for various vegetation types.

## Background

Rice and wheat are two major staple crops in the world and provide primary dietary calories and protein for the global population [1, 2]. Leaf nitrogen (N) content is an important indicator of crop photosynthetic capacity [3] and is needed by agronomists for making fertilization recommendations [4]. Quantification of leaf N content could provide valuable information for monitoring crop physiology [5], and practicing precise farming [6] so as to improve the use efficiency of nitrogen fertilizers. Remote sensing has been widely used as a non-destructive approach for estimating leaf N content in the past few decades [7–9]. The common practice is to establish linear or nonlinear relationships between leaf N content and spectral features derived from leaf reflectance spectra.

Curran [10] listed 42 absorption features caused by bending and stretching of foliar chemical bonds in the 400–2400 nm range and identified many of them related to N in the shortwave infrared region (SWIR) region (1000–2400 nm). Based on a number of N-sensitive wavelengths in the SWIR region, leaf N can be estimated accurately from the reflectance spectra of dried and ground leaves [11, 12]. However, these absorption features are masked by water absorption and hence not clearly visible in the SWIR reflectance spectra of fresh leaves, thereby leading to weaker signals of N in the entire spectra [13–15]. This has been proven by the findings that the accuracy of N estimation from the reflectance spectra of fresh leaves is lower than that of dry leaves or dried and ground leaves [11]. Therefore, the SWIR region in the reflectance spectra of fresh leaves has seldom been used for N estimation. Instead, the visible and near infrared (VNIR) region has been widely used for this purpose [8, 16, 17] because nitrogen and chlorophyll are well related [3, 18] and the latter has deep absorption valleys in this region [19, 20]. To date, most studies built N estimation models with the spectral information of fresh leaves in the VNIR region alone [21–23] or VNIR and SWIR regions [9, 24, 25]. A few studies made use of SWIR reflectance alone [12] but their focus was on the spectra of dried leaves or leaf powder, rather than fresh leaves that are easier to handle for reflectance measurements. How accurately the N content could be estimated from reflectance spectra of fresh leaves in the SWIR region alone is poorly understood.

As a group of N-containing compounds in fresh leaves, chlorophylls account for only a portion of the total nitrogen [3, 26]. The physical mechanism underlying the spectroscopic estimation of leaf N content remains unclear due to the use of indirect N-sensitive wavelengths in the VNIR region. The use of SWIR region may help us reveal the mechanism and better understand the spectral responses to N variation [10, 11]. A major problem constraining the satisfactory estimation of N content is the effect of leaf water absorption on reflectance spectra, which was claimed to be removed to within 10% by Kokaly and Clark [11]. Recently, a few studies adopted a water removal technique originally proposed by Gao and Goetz [27] to remove the effect of water absorption so as to improve the estimation of N content [15, 28] and N to phosphorous ratio [29]. These studies compared water removal to traditional spectral transformation techniques, but did not decompose the water removal process for understanding the underlying mechanism of this technique. To make use of the spectral information in the SWIR region, they applied traditional methods such as stepwise multiple linear regression and partial least-squares regression to the spectra after the water removal process. Based on these methods, some of the selected wavelengths were not related to the absorption features of the chemical being examined [12] and the regressions were often faced with model overfitting and indirect relationships [10]. In addition, how to enhance the causal absorption features in WR spectra for the improved estimation of leaf N content is poorly understood.

Recently, continuous wavelet analysis (CWA) has been widely used to estimate chlorophyll content [30–32], water content [33, 34], dry matter content [35], and leaf area index [36] from leaf and canopy reflectance spectra. After the application of continuous wavelet transform, a reflectance spectrum is decomposed into a number of scale components, which have the same length as the reflectance spectrum and are composed of wavelet features as a function of wavelength and scale [33]. Wavelet features have been proven to be superior to vegetation indices (VIs) in the characterization of absorption by foliar chemicals in reflectance spectra [33, 35, 37]. However, none of previous studies have used CWA and water removal techniques collectively and have investigated the application of CWA to WR spectra for examining dry matter and nitrogen related absorption characteristics. In contrast to the continuum removal operation used in Schlerf et al. [15] and Ramoelo et al. [28], the continuous wavelet transform (CWT) is a linear operation and enables us to decompose the CWT of WR spectra into that of spectral addition or subtraction. Combining CWA and water removal has the potential to improve the estimation of N content by reducing the effect of water absorption and enhancing N absorption signals.

Common measures for expressing leaf N content are either area-based (N_area_, g/m^2^) or mass-based (N_mass_, %). Because of its tight correlation with photosynthetic capacity [3] and the widespread use in fertilization management [38, 39], N_mass_ has been extensively studied and estimated from remotely sensed data [7, 23–25, 40]. Both N_area_ and N_mass_ can be directly obtained in a destructive way, but remote sensing usually works better for estimating N_area_. The leaf biochemistry for dried ground samples is usually expressed as concentration (mass-based, mass per unit dry weight). For intact fresh leaves, the biochemistry is often expressed as content (area-based, mass per unit leaf area) [41]. The use of chemical content may be more suitable for remote sensing applications because it is a better representation of the interaction between matter and light per unit surface area. For example, the units of biochemical parameters used in PROSPECT model are all area-based [19, 42]. It may be proven by the higher correlations of many VIs with N_area_ than N_mass_ documented in Hansen and Schjoerring [22] and Jay et al. [43]. Although the interaction of leaves with light per unit leaf area is directly related to such area-based traits as N_area_ [41, 44], few studies paid attention to the estimation of N_area_ [13, 43, 45]. As pointed out by Wright et al. [46], N_mass_ and N_area_ are interconverted via leaf mass per area (LMA, g/m^2^). To the best of our knowledge, only one study made use of this connection and derived N_area_ indirectly from estimated N_mass_ and LMA [25]. Based on our understanding of N_mass_ and N_area_, this study will investigate first estimating the two area-based factors (N_area_ and LMA) and then indirectly estimating N_mass_ instead. The SWIR region encompasses all of the major absorption features of dry matter and N and may provide sufficient spectral information to estimate LMA, N_area_ and N_mass_.

Therefore, the objectives of this study were (1) to determine the wavelet features most sensitive to LMA and N_area_ from both leaf reflectance spectra and WR spectra in the SWIR region, (2) to evaluate the feasibility of improving LMA, N_area_ and N_mass_ estimations with the integration of CWA and the water removal technique, and (3) to compare the CWA and normalization difference (ND) index approaches in the performance of LMA, N_area_ and N_mass_ estimations.

## Methods

### Experimental design

Four experiments were conducted at the experimental station in Rugao, Jiangsu of eastern China (120°45′E and 32°16′N) with two for rice and two for wheat. Both crops were chosen as they were grown in rotation in this experimental area. We intended to develop robust models for both of them despite their differences in biochemical parameters and surface properties [47]. The pooled data represented a wider range of samples and were beneficial to test the stability of the method proposed in this study. The treatment in each experiment represented variations in cultivar type, nitrogen fertilization rate and planting density with three replications. These treatments were applied to create wide ranges for N_mass_, N_area_ and LMA, which includes the extremely high and low values. There were a total of 36 plots with the same size of 5 m × 6 m for each experiment. Details about the experiments and sampling dates are shown in Table 1. Rice was transplanted and grown in a grid pattern with a plant spacing of 15 cm and two row spacings of 30 and 50 cm. Wheat was sowed in drill with different row spacings and the rows were oriented in a south-north direction. The 2-year experimental data for rice and wheat were combined to form RICE and WHEAT datasets, respectively.

**Table 1:** Summary of designs and ground samplings periods of the field plot experiments

Dataset	Year of data collection	Cultivar	Nitrogen rate (kg hm^−2^)	Planting density (cm)	Growth stages for sampling	Number of samples
Rice	2015	Yliangyou 1* Wuyunjing 24**	0, 100, 200, 300	30 × 15, 50 × 15	Jointing, booting, heading	95
	2016	Yliangyou 1* Wuyunjing 24**	0, 150, 300	30 × 15, 50 × 15	Jointing, booting	74
Wheat	2016	Yangmai 18*	0, 80, 150, 220	20, 30, 40	Jointing, booting, heading	127
	2017	Yangmai 15* Yangmai 16**	0, 150, 300	25, 40	Late-jointing, heading, anthesis	104

*, **Denote erect-leaf and drooping-leaf cultivars, respectively

### Measurements of reflectance spectra and chemical constituents

Three leaves per plot were collected and each leaf made a sample. Their reflectance spectra were measured using an ASD FieldSpec Pro spectrometer (Analytical Spectral Devices, Boulder, CO, USA) assisted with a leaf clip accessory. Reflectance spectra were taken per leaf for three leaf positions and these spectra were averaged to represent the leaf sample spectrum. The spectrometer collects data at 1.4 nm and 2 nm sampling interval in the 350–1000 nm and 1000–2500 nm spectral regions, respectively. The spectral data were obtained at a 1 nm spectral interval.

After collection of reflectance spectra, the surface area (A, m^2^) and fresh weight (FW, g) were measured immediately for every leaf sample. The leaf area (A, m^2^) was determined with a HP G4050 scanner (HP Development Company, L.P., USA) and calculated as the product of pixel count per leaf and the actual area represented by each pixel. The leaf dry weight (DW, g) was measured after the leaves were dried in the oven at 80 °C for 48 h. Finally, equivalent water thickness (EWT, g/m^2^), leaf mass per area (LMA, g/m^2^) and area-based leaf nitrogen content (N_area_, g/m^2^) were calculated with the equations below:

1 $${\text{EWT}} = \left( {{\text{FW}} - {\text{DW}}} \right)/{\text{A}}$$

2 $${\text{LMA}} = {\text{DW}}/{\text{A}}$$

3 $${\text{N}}_{\text{area}} = {\text{N}}_{\text{mass}} \times {\text{LMA}}$$

where mass-based leaf nitrogen content (N_mass_, %) was determined using Kjeldahl method with SEAL AutoAnalyzer 3 HR (SEAL Analytical, Ltd., German). Note that the unit g/m^2^ of EWT can be interconverted to cm (1 g/m^2^ = 10^−4^ g/cm^2^ = 10^−4^ cm) via the density of water (1 g/cm^3^: 1 g water = 1 cm^3^ water) [48].

### The adjusted water-removal technique

The water removal technique was originally proposed by Gao and Goetz [27] with the assumption that there is a linear spectral background level and a nonlinear combination of a reflectance spectrum of leaf water and that of leaf dry matter for fresh leaves. However, the assumption of linear spectral background is only valid for small wavelength regions [27] and this technique is often performed on spectral segments, such as 1500–1780 nm and 2100–2200 nm [15, 27]. The use of narrow and discrete spectral segments may limit the utility of full-range spectral information. Therefore, this technique was adjusted in this study to obtain full-range water removed (WR) spectra by adopting PROSPECT-5B [42] for modeling leaf reflectance spectra. The input parameters of PROSPECT-5B include chlorophyll content (C_*ab*_), carotenoid content (C_*xc*_), EWT, LMA, and structural parameter (*N*_*struc*_) [42]. To estimate the WR spectra, the first step was to find the best combination of input parameters for PROSPECT-5B by minimizing the following merit function:

4 $$min = \mathop \sum \limits_{\lambda = 1}^{n} \left( {R_{\lambda } - \widetilde{{R_{\lambda } }}} \right)^{2}$$

where *R*_*λ*_ and $\widetilde{{R_{\lambda } }}$ are the measured reflectance (MR) and modeled reflectance values of a spectrum at wavelength *λ*, respectively. The mathematical optimization was performed with the ‘CONSTRAINED_MIN’ function in IDL 8.3 (Exelis Visual Information Solutions, Boulder, CO, USA), which gives the best combination of parameters that minimizes the difference between modeled and measured reflectance for the specified spectral range. Since most nitrogen and dry matter related absorption features are located in the shortwave infrared (SWIR) region [10], the spectral range 1000–2400 nm was used in this study for implementing PROSPECT model inversion as well as the determination of WR spectra. In addition, the *C*_*ab*_ and *C*_*xc*_ were fixed at 30 and 3 μg/cm^2^, respectively, because these pigments have no absorption in this spectral region [42, 47].

After the PROSPECT model inversion process, the second step was to model the leaf water reflectance spectra by inputting the inverted parameters into PROSPECT-5B with zero LMA values. Since pigments have no influences on the reflectance in this spectral region, the parameters C_ab_ and C_xc_ were fixed at 30 and 3 μg/cm^2^, respectively [42, 47]. As water was the only absorbing chemical left in fresh leaves for this purpose, the modeled leaf water reflectance spectra were named as water-only (WO) spectra. The residual spectra, namely the WR spectra, were estimated as below:

5 $$WR_{\lambda } = \left( {R_{\lambda } - R_{\lambda }^{wo} } \right)/\bar{R}$$

where *R*_*λ*_^*wo*^ was the reflectance of a WO spectrum at wavelength λ. $\bar{R}$ was the mean reflectance of a MR spectrum over all wavelengths.

**Fig. 1:**
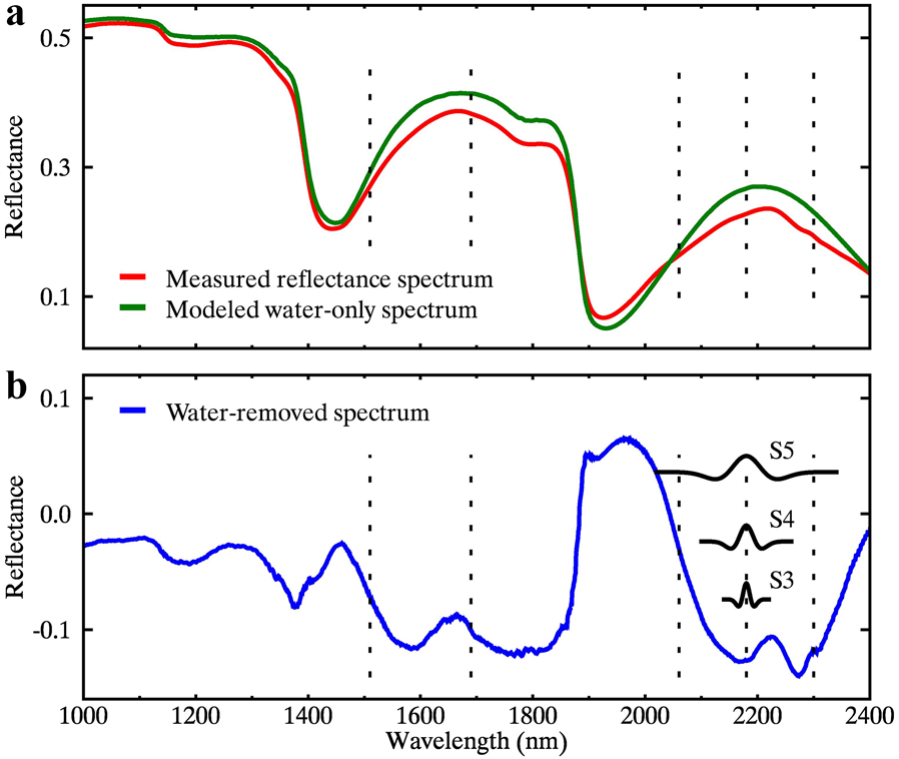
**a** Represents a measured reflectance spectrum and the modeled water-only spectrum for a fresh leaf and **b** represents the corresponding water-removed spectrum. The vertical dashed lines correspond to the absorption features of nitrogen centered at 1510 nm, 1690 nm, 2060 nm, 2180 nm, and 2300 nm. In addition, three scaled and shifted mother wavelets (labeled as S3, S4, and S5) at scales 3–5 with the shifting factor of 2180 nm are presented in (**b**)

After the absorption of dry matter was removed, the amplitude of modeled leaf water reflectance increased for all wavelengths except for the strong water absorption region around 1900 nm (Fig. 1a). Many nitrogen-related absorption features that were not obvious in the MR spectrum became visible in the WR spectrum, such as the feature at 2180 nm (Fig. 1b). Note that the reflectance values have physical meanings and cannot be negative, but the amplitudes of WR spectra were relative values estimated from MR spectra and the negative MR values only meant the negative differences between a reflectance spectra and the corresponding water-only spectrum. Similar terminology is also seen in “derivative spectra”, which represents the derivative data of reflectance spectra and encompasses negative and positive values [49].

### Continuous wavelet analysis (CWA)

Mathematically, continuous wavelet transform (CWT) is a linear operation that performs the convolution of reflectance spectrum with a scaled and shifted mother wavelet. The realization process is shown as below:

6 $$\psi_{a,b} \left( {{\uplambda }} \right) = \frac{1}{\sqrt a }\psi \left( {\frac{\lambda - b}{a}} \right)$$

7 $$W_{r} ( {a,b}) = \int\limits_{- \infty }^{+\infty } r(\lambda )\psi_{a,b} ( \uplambda)d\lambda$$

where $\psi \left( {{\uplambda }} \right)$ is the mother wavelet function and $\psi_{a,b} \left( {{\uplambda }} \right)$ is the scaled and shifted version of $\psi \left( {{\uplambda }} \right)$. *W*_*r*_(*a*, *b*) is the wavelet coefficient (or wavelet feature, denoted as WF_b,a_) for the scaling factor *a* and the shifting factor *b*. The scaling factor indicates the width of the scaled mother wavelet, which can be comparable with the width of an absorption feature. Narrow absorption features can be captured by a low scaling factor while broad features need high scaling factors. The scaling factor used in this study is at dyadic scales 2^3^ (scale 3), 2^4^ (scale 4), 2^5^ (scale 5), and 2^6^ (scale 6). The shifting factor determines the central wavelength of shifted mother wavelet, which is used to capture the peak or valley of an absorption feature. Taking the absorption feature of nitrogen at 2180 nm as an example, the three scaled and shifted mother wavelets (Scale 3, Scale 4, and Scale 5) are displayed in Fig. 1b. The spectral width of the corresponding wavelet feature increases with the scaling factor. Different with a spectral index calculated with two discrete wavelengths, a wavelet feature represents the information for a continuous spectral sub-region that determined by scaling and shifting factors. Although CWA is known to be resistant to signal noise, noise reduction is not the focus of this study. Rather, we took the advantage of CWA in capturing and enhancing the absorption features by nitrogen and dry matter. The multiscale property of CWA is well suited for characterizing the absorption changes that may occur in different widths over different wavelength ranges.

The one-dimensional reflectance spectra are converted into two-dimensional wavelet power (magnitude of wavelet coefficient) datasets after the application of CWT. Hence, a scalogram of coefficients of determination (*R*^2^) was obtained at all wavelengths and scales [33]. Moreover, the regions that compromised the top 1% *R*^2^ values were extracted from the comprised scalograms and finally the wavelet feature with the highest *R*^2^ was selected. In this study, the mother wavelet function was determined as the second derivative of the Gaussian function because of its similarity to the shapes of leaf absorption features [35]. CWT was conducted using the ‘WV_CWT’ function in IDL 8.3 (Exelis Visual Information Solutions, Boulder, CO, USA). Since the wavelet is linearly additive [37], the wavelet power derived from a WR spectrum equals the difference in wavelet power between the MR spectrum and the WO spectrum divided by the mean of reflectance over all wavelengths as follows:

8 $$WP(WR_{\lambda } ) = WP\left( {R_{\lambda } )/\bar{R} - WP(R_{\lambda }^{wo} } \right)/\bar{R}$$

where WP represents the wavelet power and other symbols are the same as in Eq. (5).

### Evaluation of estimation accuracy

The widely used normalization difference (ND) indices were calculated in this study for comparison with the wavelet features in the evaluation of estimation accuracy. The ND index wavelengths were determined as the best band combination from *R*^2^ scalograms except these combinations with difference between two wavelengths within 10 nm [50, 51]. Since the aim of this study was to build a robust model across rice and wheat crops, the most sensitive wavelet features and ND indices to LMA, N_area_ and N_mass_ were determined separately from pooled data. These spectral features were used to represent their regression relationships and generate direct estimates of those traits. Since area-based foliar traits are directly related to the interaction of foliar constituents with light and can be more easily estimated with spectral features than mass-based traits [41, 44], N_mass_ was also estimated in an indirect way as the ratio of estimated LMA and N_area_ values as below:

9 $${\text{Estimated }}\,{\text{N}}_{\text{mass}} = \frac{{{\text{Estimated }}\,{\text{N}}_{\text{area}} }}{{{\text{Estimate}}\, {\text{LMA}}}} \times 100{{\% }}$$

The indirect estimation of N_mass_, based on two wavelet features separately sensitive to N_area_ and LMA, might outperform the direct estimation with a single wavelet feature.

The estimation accuracies were evaluated with the metrics *R*^2^ and root mean square root (RMSE):

10 $$R^{2} = 1 - \frac{{\mathop \sum \nolimits_{i} \left( {y_{i} - y_{i}^{\prime } } \right)^{2} }}{{\mathop \sum \nolimits_{i} \left( {y_{i} - \bar{y}} \right)^{2} }}$$

11 $$RMSE = \sqrt {\frac{{\mathop \sum \nolimits_{i} \left( {y_{i} - y_{i}^{\prime } } \right)^{2} }}{n}}$$

where *y*_*i*_ and $y_{i}^{\prime }$ are the measured and estimated trait values for sample *i*. $\bar{y}$ is the arithmetic mean of trait and n is the number of samples. Due to the intrinsic discrepancy between rice and wheat leaves, the best feature for rice may not be exactly the same as that for wheat and vice versa. A generic model that would work best across rice and wheat samples was preferred over that specific to one crop alone. Therefore, the selected features were determined from the pooled data of rice and wheat. These evaluation metrics were calculated from two commonly used validation processes. One is a 10-fold cross validation procedure applied to the pooled data, and the other is the leave-one-out (LOO) method. In the LOO process, three sub-datasets were used for model calibration and the left one was used for validation. This validation process was repeated for the every one of the four sub-datasets (Rice 2015, Rice 2016, Wheat 2016, and Wheat 2017) in Table 1. Since many studies also used the VNIR region to calculate the Red-Edge Chlorophyll Index (CI_red edge_) for leaf N content estimation [16, 52], a brief comparison between SWIR and VNIR regions was presented.

## Results

### Descriptive statistics of foliar traits

**Fig. 2:**
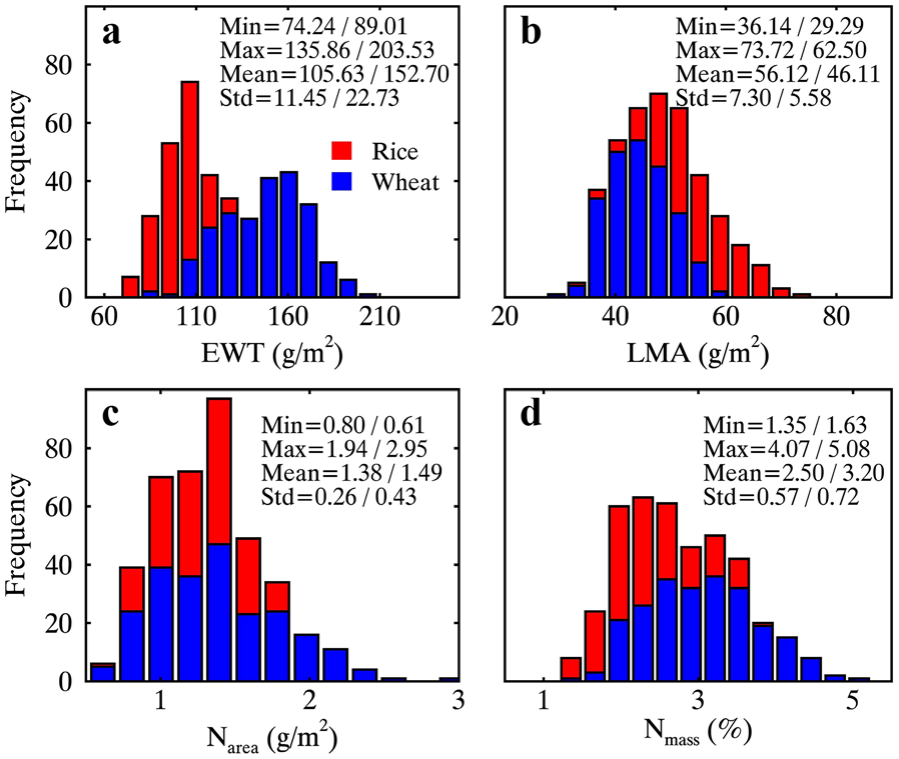
Stacked frequency distributions of **a** EWT, **b** LMA, **c** N_area_, and **d** N_mass_ for the two datasets RICE and WHEAT. The statistics before and after slash (/) in each distribution plot are shown for RICE and WHEAT, respectively

Differences in foliar traits between the two datasets could be observed in terms of range and magnitude (Fig. 2). Compared with RICE, the WHEAT dataset exhibited a higher mean and a wider range for EWT, N_area_ and N_mass_ (*p* < 0.01) but a lower mean and a narrower range for LMA (*p* < 0.01). In addition, N_area_ was positively correlated with N_mass_ for both RICE (*r* = 0.79, *p* < 0.01) and WHEAT (*r* = 0.92, *p* < 0.01) (Table 2). N_area_ was positively correlated with LMA for WHEAT (*r* = 0.65, *p* < 0.01) but not correlated with EWT for both datasets. N_mass_ was correlated with LMA for RICE (*r* = − 0.56, *p* < 0.01) and WHEAT (*r* = 0.31, *p* < 0.01) but correlated with EWT only for RICE (*r* = − 0.30, *p* < 0.01).

**Table 2:**
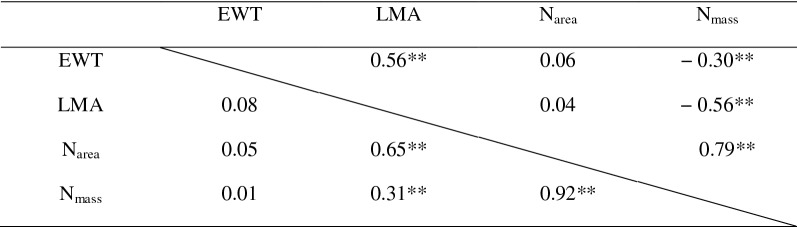
Pearson’s correlation matrix for EWT, LMA, N_area_ and N_mass_ (top-right triangle for RICE and bottom-left triangle for WHEAT)

**Significant correlations with *p* < 0.01

**Fig. 3:**
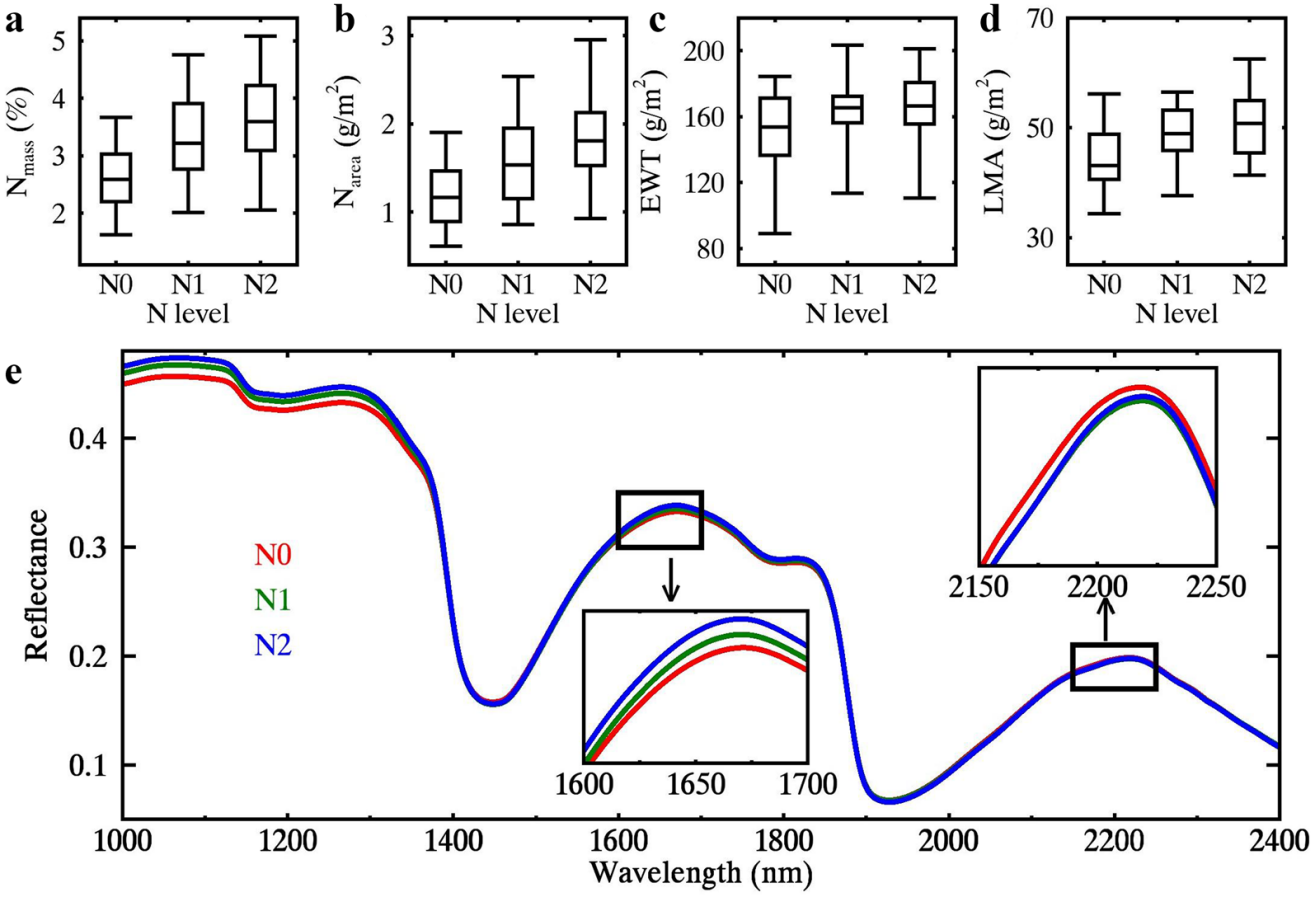
The boxplots of **a** N_mass_, **b** N_area_, **c** EWT and **d** LMA as well as **e** averaged leaf reflectance spectra under three nitrogen treatments (N0: 0 kg hm^−2^, N1: 150 kg hm^−2^, N2: 300 kg hm^−2^) for WHEAT 2017 samples

As one of the most important factors in this experiment, the effect of N treatment on leaf reflectance spectra is displayed in Fig. 3 by taking the WHEAT 2017 samples as an example. The N_mass_, N_area_, and LMA values for WHEAT 2017 increased with the N fertilization rate, and EWT was almost invariant for N1 and N2 (Fig. 3a–d). The leaf reflectance spectra under three N treatments exhibited marginal differences in the SWIR region (Fig. 3e). This phenomenon was expected for fresh leaves, which demonstrated the need of water removal technique to remove the water absorption and CWA to enhance the absorption features of nitrogen and dry matter.

### Relationships of N_area_ with optimal wavelet features and ND indices

**Fig. 4:**
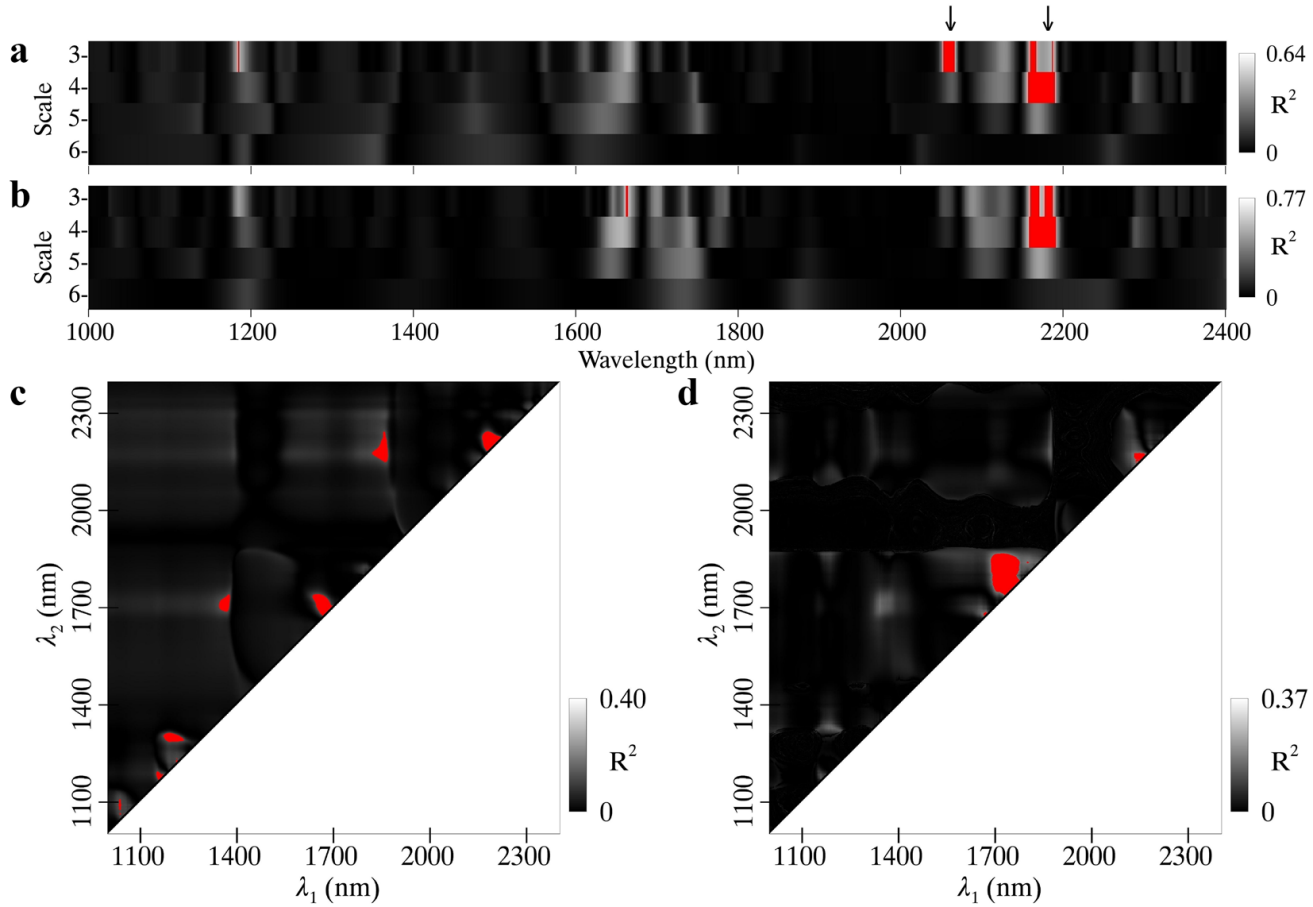
Coefficients of determination (*R*^2^) for the relationships of N_area_ with **a** MR spectra and **b** WR spectra derived wavelet features, and **c** MR spectra and **d** WR spectra derived ND indices. Regions highlighted in red on the scalograms represent the top 1% *R*^2^ for wavelet features or ND indices. The downward arrows on the top of the figure indicate the wavelength locations of absorption features with both 2060 nm and 2180 nm for protein and nitrogen

The highest *R*^2^ values between N_area_ and wavelet features for pooled data were 0.64 and 0.77 for MR and WR spectra, respectively (Fig. 4a, b). The two feature regions determined for MR spectra were centered at 2060 nm and 2180 nm, and both of them matched up with the absorptions of protein and nitrogen [10]. As for the WR spectra, a similar feature region with longer wavelengths was found, and a minor one exhibited much shorter wavelengths than the counterpart for the MR spectra.

The *R*^2^ contour maps for the relationship between N_area_ and ND indices are shown in Fig. 4c, d for MR (maximum *R*^2^ = 0.40) and WR (maximum *R*^2^ = 0.37) spectra, respectively. Seven wavelength regions most sensitive to N_area_ were observed for the MR spectra and five of them were close to the diagonal line, which indicates the presence of similar wavelengths in an ND index. As for the WR spectra, the majority of wavelength combinations were located in the 1700–1800 nm region in which both *λ*_*1*_ and *λ*_*2*_ fall.

**Fig. 5:**
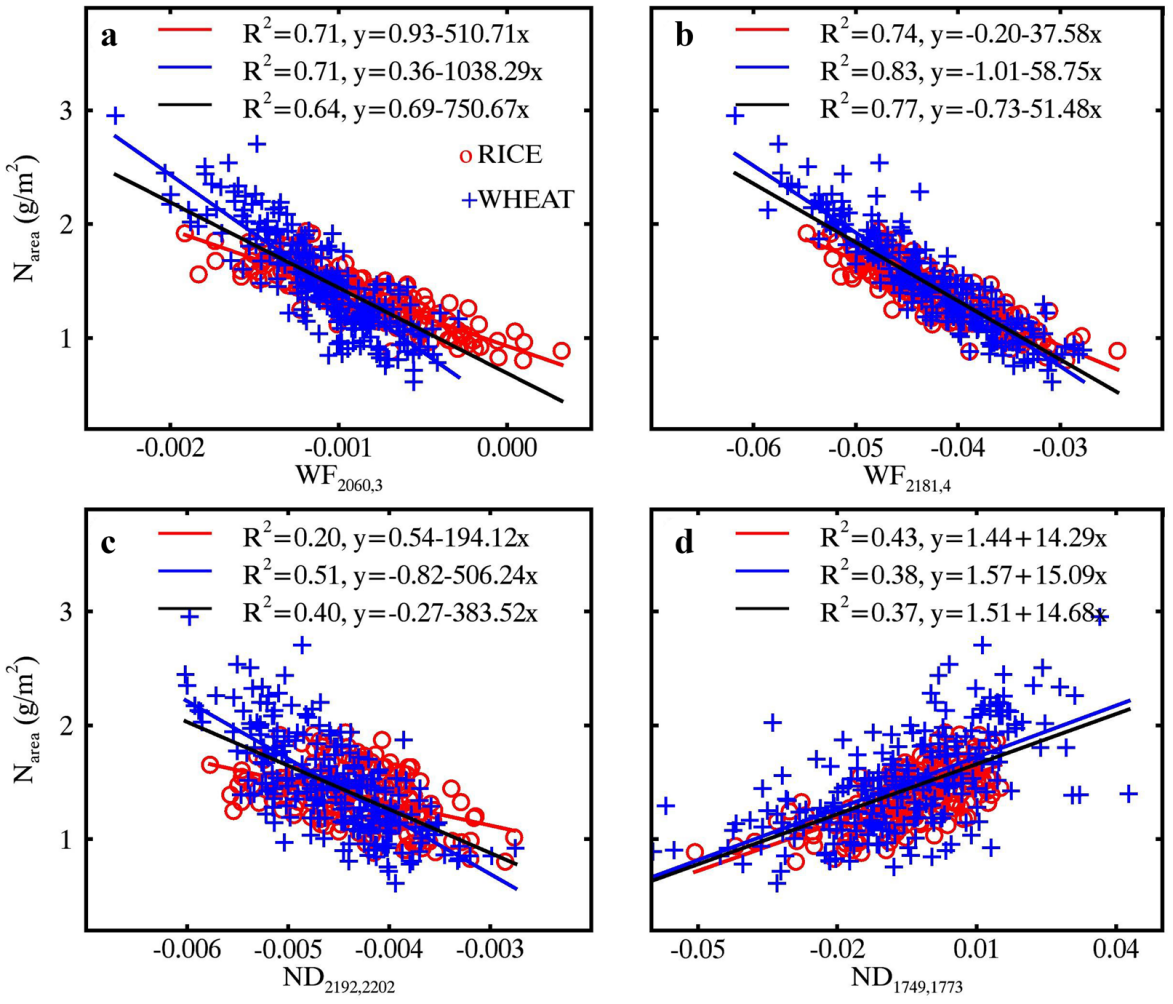
Relationships of N_area_ with wavelet features (top row) and ND indices (bottom row) derived from MR spectra (left column) and WR spectra (right column). Red, blue and black lines represent linear fits on RICE, WHEAT and pooled data, respectively. All regressions are statistically significant (*p *< 0.001)

Figure 5 shows the relationships of N_area_ with four optimal spectral features (WF_2060,3_, WF_2181,4_, ND_2192,2202_ and ND_1749,1773_) determined from the top 1% correlations in Fig. 4. The two optimal wavelet features (WF_2060,3_ and WF_2181,4_) used the spectral information over two spectral sub-regions that matched well with the absorption features centered at 2060 nm and 2180 nm (Fig. 4a, b). Nevertheless, the two optimal ND indices (ND_2192,2202_ and ND_1749,1773_) used the spectral information at four individual wavelengths 1749 nm, 1773 nm, 2192 nm and 2202 nm (Fig. 4c, d). All of the relationships for WHEAT, RICE and pooled data were linear. After the application of the WR process, the N_area_ ~ WF correlation exhibited a pronounced improvement (*R*^2^ increased from 0.71 to 0.83) over that from the MR spectra for the WHEAT dataset but a slight increase for RICE dataset (Fig. 5a, b). Overall, this caused an increase of *R*^2^ from 0.64 to 0.77 for the pooled data. For N_area_ ~ ND correlations, the combination of an increase for RICE and a decrease for WHEAT resulted in an overall decrease in *R*^2^ (Fig. 5c, d).

**Fig. 6:**
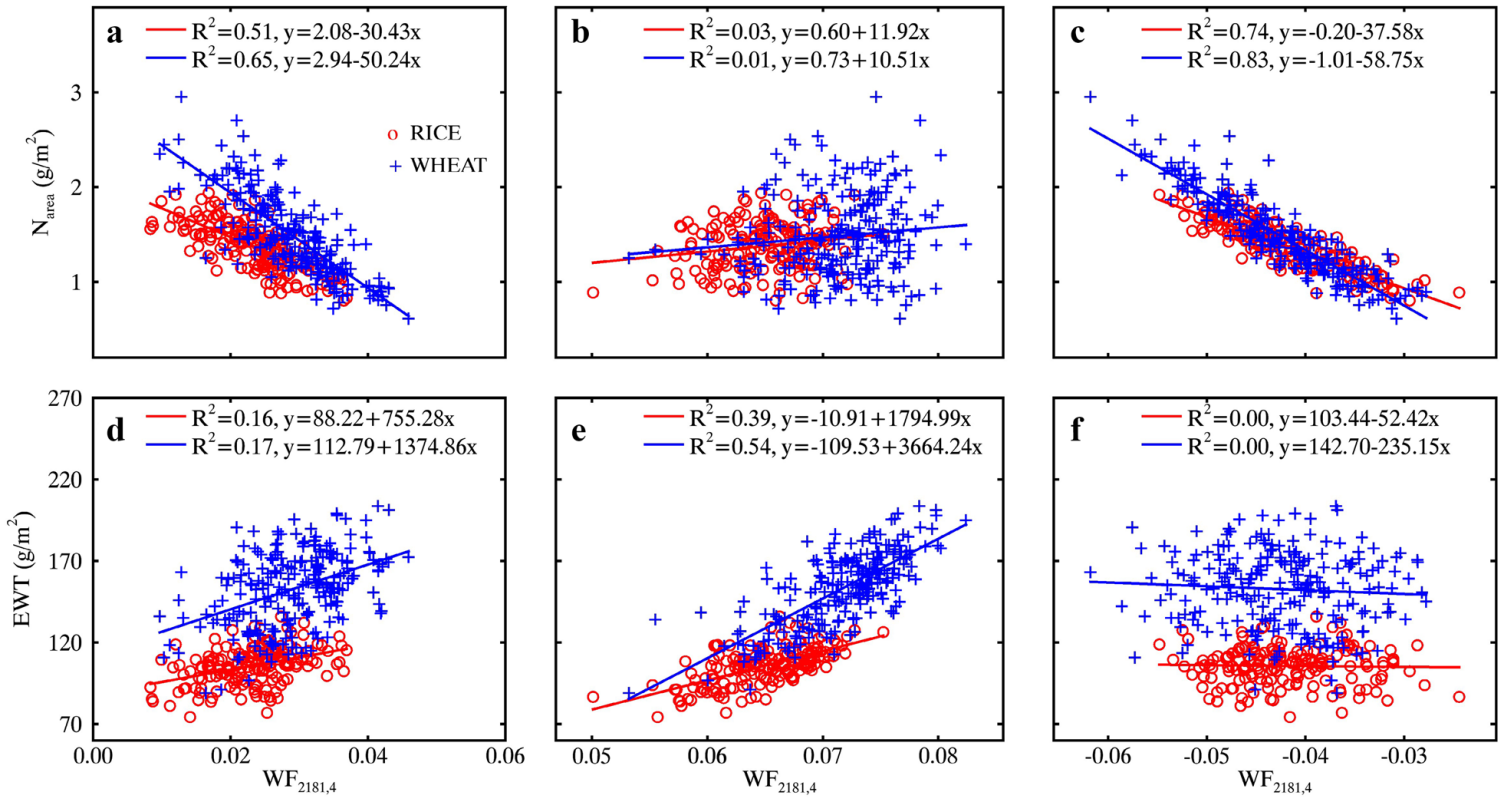
Relationships between WF_2181,4_ and (**a**, **b**, **c**) N_area_ and (**d**, **e**, **f**) EWT derived from MR spectra (left column), WO spectra (middle column) and WR spectra (right column). Particularly, the WF_2181,4_ at right column was equal to the difference between WF_2181,4_ at left column and middle column. Red and blue lines represent linear fits on RICE and WHEAT, respectively. All regressions are statistically significant (*p* < 0.001) except for that in (**b**, **f**)

Because of the linearly additive property of CWT, the final regressions from the WR spectra could be decomposed into intermediate regressions to understand how the effect of water absorption was removed. Taking the N_area_ estimation as an example, Fig. 6 shows the intermediate and final regressions based on Eq. (8) to illustrate the advantages of the water removal technique. N_area_ was well correlated to the WF_2181,4_ derived from the MR spectra (Fig. 6a) but not to the WF_2181,4_ derived from the WO spectra for both RICE and WHEAT datasets (Fig. 6b). The WF_2181,4_ ~ N_area_ relationship in the MR spectra was weaker than that in the WR spectra (Fig. 6c, d). After the removal of water absorption information in the WO spectra (Fig. 6e), the WR-derived WF_2181,4_ that equaled the difference between MR-derived and WO-derived WF_2181,4_ values contained no more water information (*p* < 0.001) (Fig. 6f). Clearly, the WR-derived WF_2181,4_ showed improved relationships with N_area_ with increases of *R*^2^ values by 0.23 and 0.18 for RICE and WHEAT datasets, respectively.

### Relationships of N_mass_ with optimal wavelet features and ND indices

**Fig. 7:**
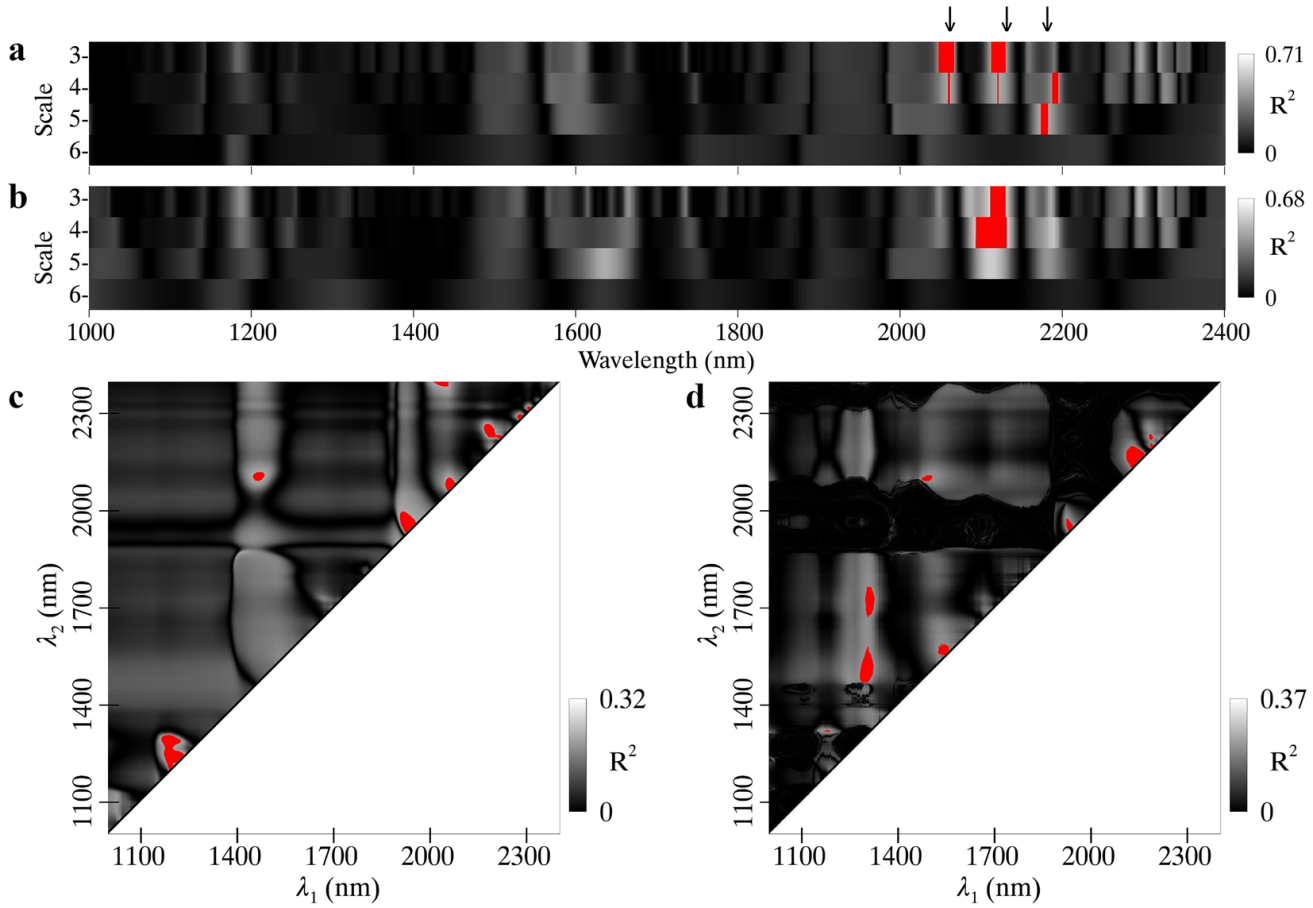
Coefficients of determination (*R*^2^) for the relationships of N_mass_ with **a** MR spectra and **b** WR spectra derived wavelet features and **c** MR spectra and **d** WR spectra derived ND indices. Regions highlighted in red on the scalograms represent the top 1% *R*^2^ for wavelet features or ND indices. The downward arrows on the top of the figure indicate the wavelength locations of absorption features with both 2060 nm and 2180 nm for protein and nitrogen and 2130 nm for protein

The highest *R*^2^ between N_mass_ and wavelet features for pooled data were 0.71 and 0.68 for MR and WR spectra, respectively (Fig. 7a, b). The three feature regions determined for MR spectra were centered at 2050 nm, 2120 nm and 2170 nm, all of which corresponded to the absorption wavelengths of protein and nitrogen [10] but showed an offset about 10 nm. As for the WR spectra, only one feature region sensitive to N_mass_ was found and its center wavelength (2110 nm) was 20 nm off the known absorption feature.

The *R*^2^ contour maps for the relationships between N_mass_ and ND indices are shown in Fig. 7c, d for MR (maximum *R*^2^ = 0.32) and WR (maximum *R*^2^ = 0.37) spectra, respectively. Regardless of MR and WR spectra, wavelength regions sensitive to N_area_ were dispersed on the contour map and a majority of them were close to the diagonal line.

**Fig. 8:**
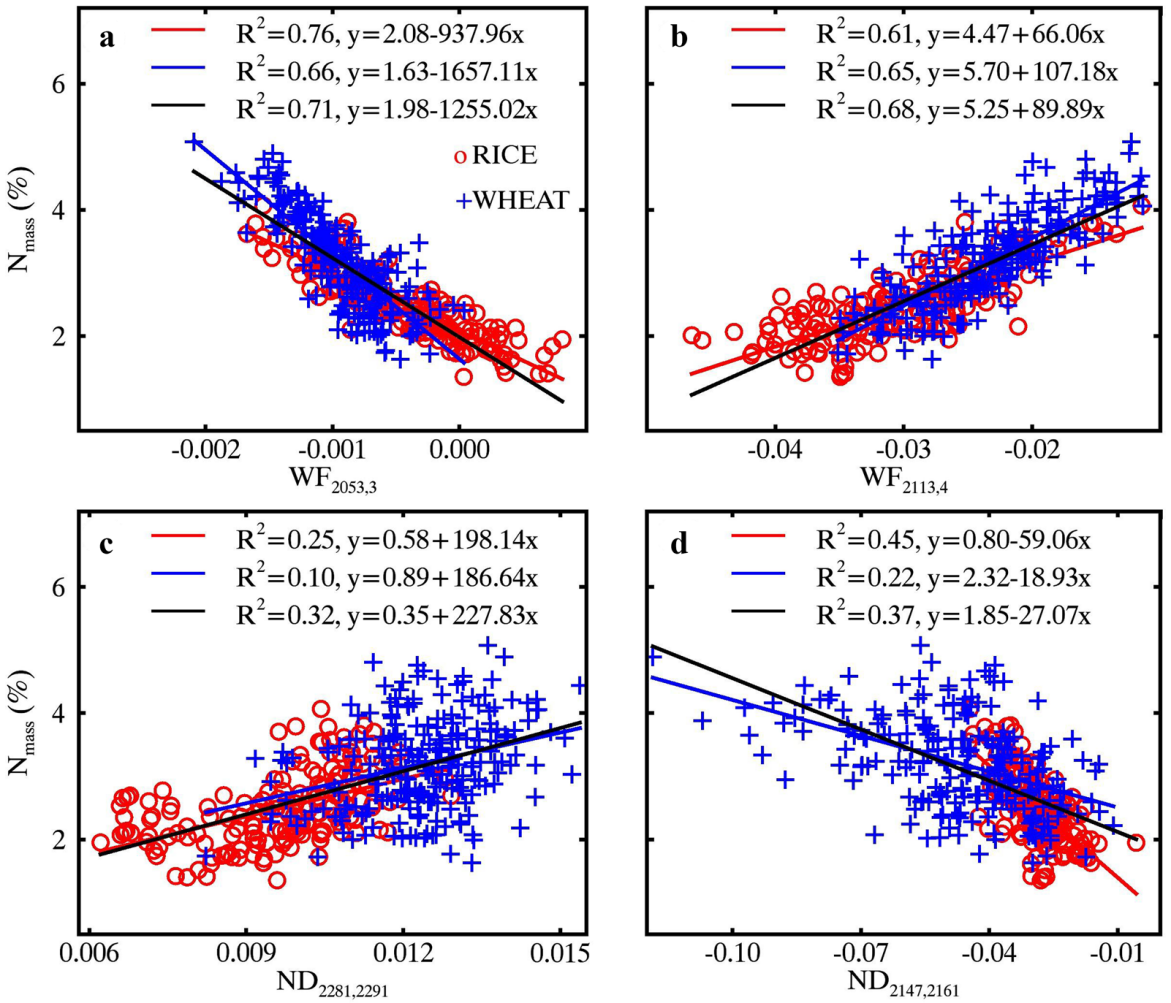
Relationships of N_mass_ with (**a**, **b**) wavelet features and (**c**, **d**) ND indices derived from MR spectra (left column) and WR spectra (right column). Red, blue and black lines represent linear fits on RICE, WHEAT and pooled data, respectively. All regressions are statistically significant (*p *< 0.001)

Figure 8 shows the linear relationships of N_mass_ with four optimal spectral features (WF_2053,3_, WF_2113,4_, ND_2281,2291_ and ND_2147,2161_) determined from the top 1% regions in Fig. 7. With the WR technique, the N_mass_ ~ WF correlation exhibited a decrease over that from the MR spectra for the individual datasets and for the pooled data (Fig. 8a, b). N_area_ ~ ND correlations increased from MR spectra to WR spectra but the *R*^2^ values were still substantially lower than those for the N_mass_ ~ WF correlations (Fig. 8c, d).

### Relationships of LMA with optimal wavelet features and ND indices

**Fig. 9:**
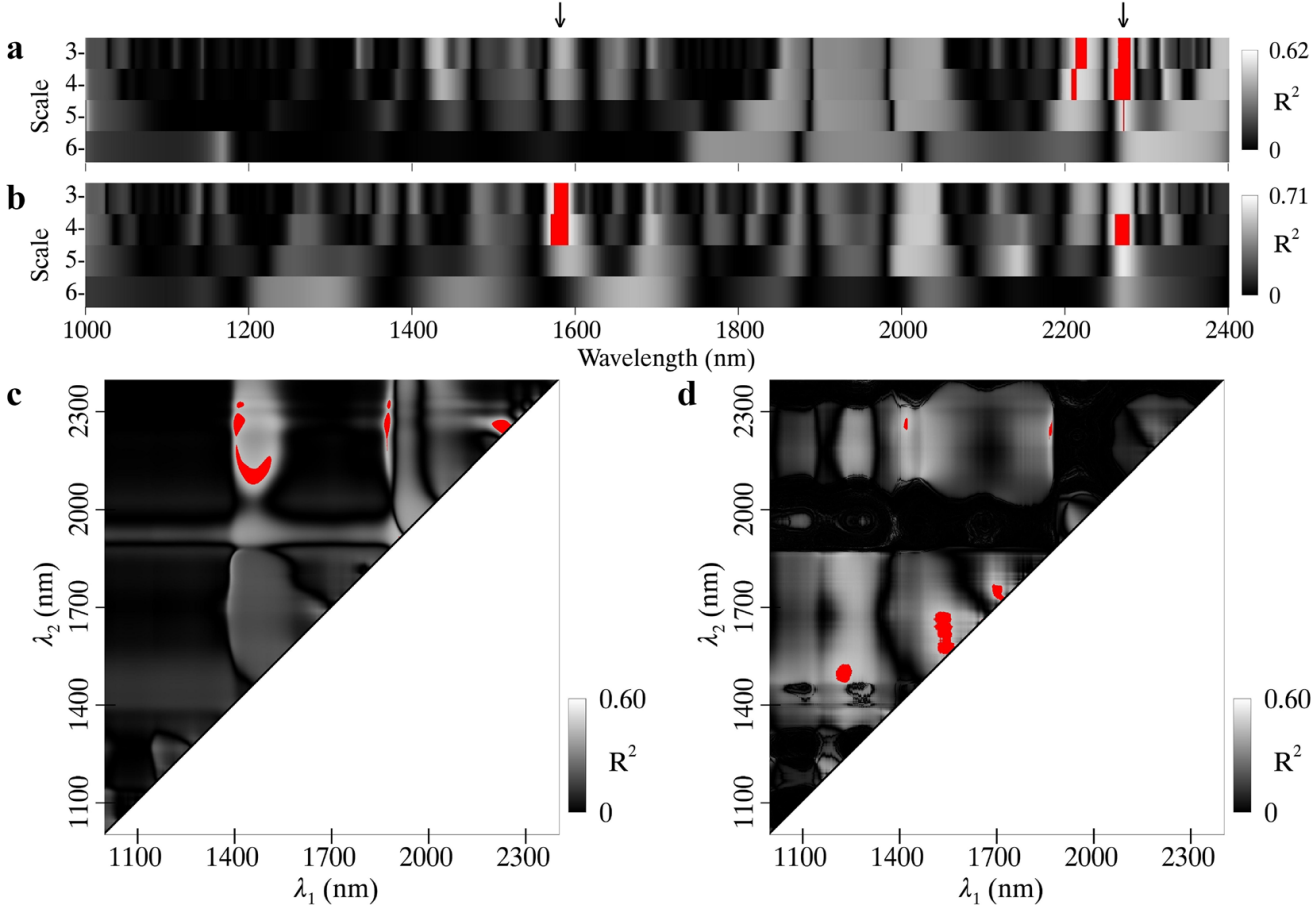
Coefficients of determination (*R*^2^) for the relationships of LMA with **a** MR spectra and **b** WR spectra derived wavelet features and **c** MR spectra and **d** WR spectra derived ND indices. Regions highlighted in red on the scalograms represent the top 1% *R*^2^ for wavelet features or ND indices. The downward arrows on the top of the figure indicate the wavelength locations of absorption features reported in [10] with 1580 nm for starch and sugar and 2270 nm for cellulose, sugar and starch

The highest *R*^2^ between LMA and wavelet features for pooled data were 0.62 and 0.71 for MR and WR spectra, respectively (Fig. 9a, b). The two feature regions determined for MR spectra were centered at 2210 nm and 2270 nm. The former did not coincide with any known absorption feature but the latter matched up with the absorption features of cellulose, sugar and starch [10]. After the water removal process, a similar feature region was found with longer wavelengths and a narrower range of scales. In addition, a new feature region with shorter wavelengths was found and exhibited a wavelength centered at 1580 nm that was associated with the absorptions of starch and sugar [10].

The *R*^2^ contour maps for the correlations between LMA and ND indices for pooled data are shown in Fig. 9c, d for MR (maximum *R*^2^ = 0.60) and WR (maximum *R*^2^ = 0.60) spectra, respectively. Three wavelength regions most sensitive to LMA for the MR spectra were represented by center wavelengths *λ*_1_ at 1450 nm, 1870 nm and 2230 nm, and *λ*_2_ at 2240 nm. As for the WR spectra, the most significant wavelength combinations were located in the regions with both *λ*_1_ and *λ*_2_ shorter than 1800 nm. The ND index regions representing the top 1% *R*^2^ with LMA were generally more scattered than the wavelet feature regions, and therefore more difficult to relate with major absorption features documented in the literature.

**Fig. 10:**
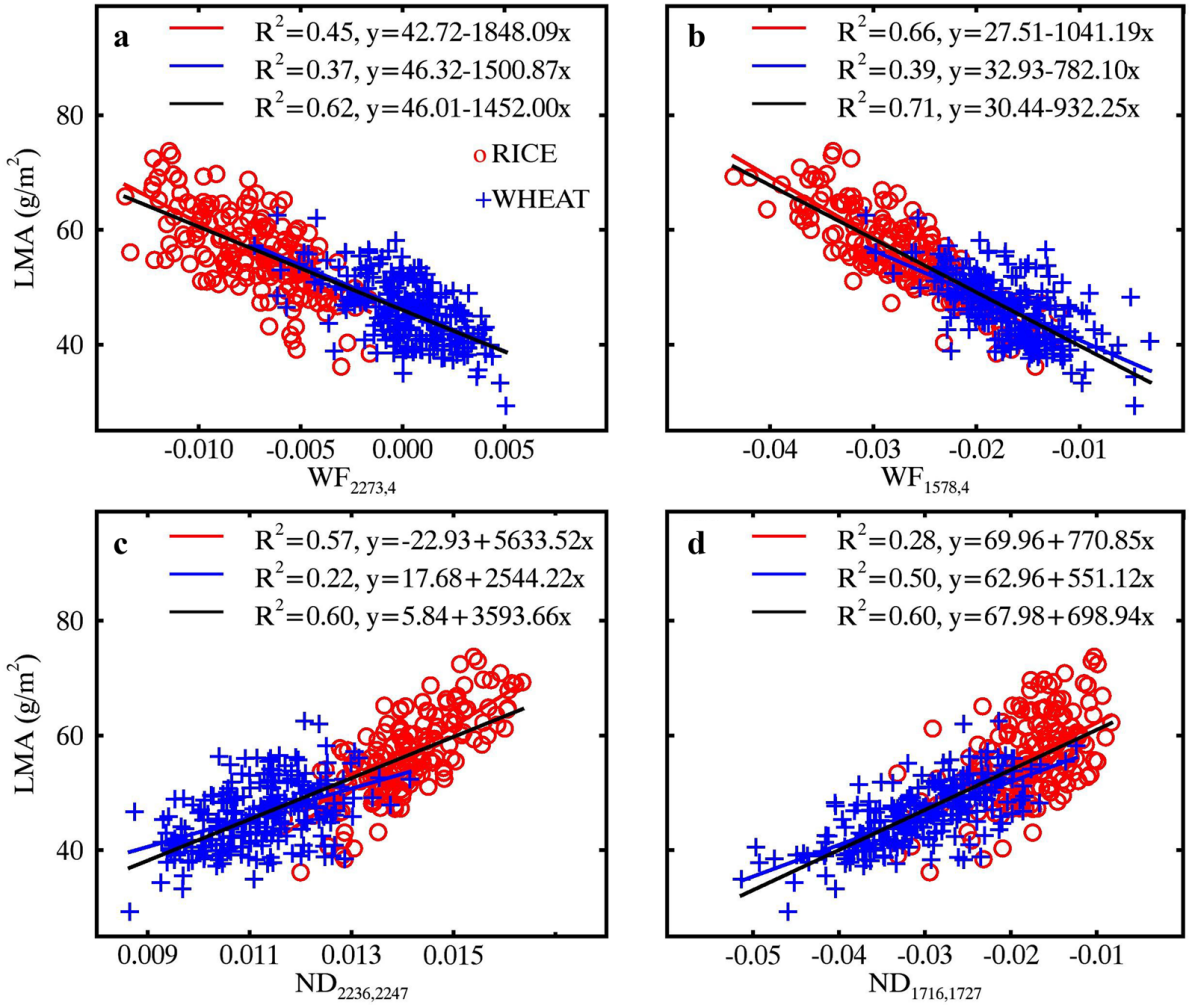
Relationships of LMA with (**a**, **b**) wavelet features and (**c**, **d**) ND indices derived from MR spectra (left column) and WR spectra (right column). Red, blue and black lines represent linear fits on RICE, WHEAT and pooled data, respectively. All regressions are statistically significant (*p *< 0.001)

Figure 10 shows the relationships of LMA with four optimal spectral features (WF_2273,4_, WF_1578,4_, ND_2236,2247_ and ND_1716,1727_) determined as the best candidates from the top 1% correlations in Fig. 9. These relationships were linear for WHEAT, RICE and pooled data. With the WR technique, the LMA ~ WF correlation exhibited a pronounced improvement (*R*^2^ increased from 0.45 to 0.66) over that from the MR spectra for the RICE dataset but did not change for WHEAT (Fig. 10a, b). This caused an increase of *R*^2^ from 0.62 to 0.71 for the pooled data. For LMA ~ ND correlations, the combination of a decrease for RICE and an increase for WHEAT led to no change in *R*^2^ for the pooled data (Fig. 10c, d).

### Assessment for the estimations of LMA, N_area_ and N_mass_

**Fig. 11:**
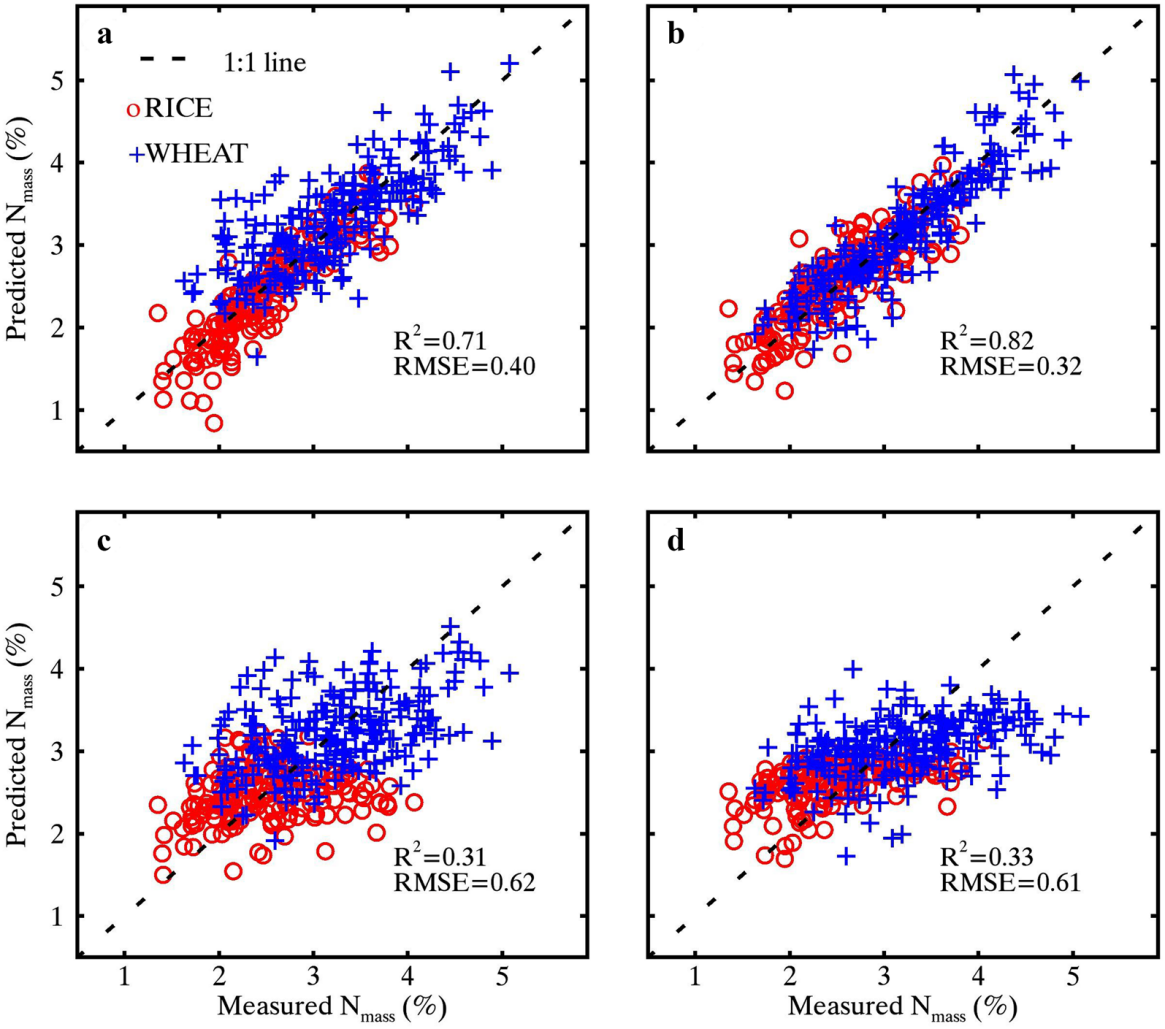
The scatter plots of measured and estimated N_mass_ derived as the ratio of LMA and N_area_ estimated from (**a**, **b**) wavelet features and (**c**, **d**) ND indices from MR spectra (leaf column) and WR spectra (right column). The LMA and N_area_ estimations were generated from the 10-fold cross validation process

Table 3 shows the estimation accuracy for LMA, N_area_ and N_mass_ over the pooled data with the 10-fold cross-validation. In particular, N_mass_ was estimated with the determined wavelet features as shown in Fig. 10 (the direct way) and with the ratio of estimated N_mass_ and LMA values (the indirect way). In all cases, wavelet features outperformed ND indices substantially in the estimations of N_area_ and N_mass_ for both MR and WR spectra. For the estimation of N_area_, the accuracy with the best correlated wavelet features was improved from MR spectra (*R*^2^ = 0.64 and RMSE = 0.22 g/m^2^) to WR spectra (*R*^2^ = 0.77 and RMSE = 0.18 g/m^2^), but that with ND indices even decreased slightly. For the direct estimation of N_mass_ with the best correlated wavelet features and ND indices, there was no significant difference in estimation accuracy before and after the application of water removal. As the ratio of wavelet-derived N_area_ and LMA, N_mass_ could be better estimated from WR spectra (*R*^2^ = 0.82 and RMSE = 0.32%) than from MR spectra (*R*^2^ = 0.71 and RMSE = 0.40%). As can be seen in Fig. 11, the scatter plot of estimated versus measured N_mass_ is closer to the 1:1 line, especially for samples in the WHEAT dataset. This indirect estimation of N_mass_ from WR spectra was also more accurate than the direct estimation. The improvement in the indirect estimation of N_mass_ from MR spectra to WR spectra was not seen for ND indices. The performance of these wavelet features derived from the SWIR region (*R*^2^ = 0.77 and 0.82 for N_area_ and N_mass_, respectively) was better than CI_red edge_ from the VNIR region (*R*^2^ = 0.76 and 0.71 for N_area_ and N_mass_, respectively), especially for N_mass_.

**Table 3:** Assessment of LMA, N_area_ and N_mass_ estimations generated with the direct and indirect ways

Dependent variables	Explanatory variables	MR spectra		Explanatory variables	WR spectra	
		*R* ^2^	RMSE		*R* ^2^	RMSE
LMA	WF_2273,4_	0.62	4.95	WF_1578,4_	0.70	4.38
	ND_2236,2247_	0.60	5.12	ND_1716,1727_	0.59	5.13
N_area_	WF_2060,3_	0.64	0.22	WF_2181,4_	0.77	0.18
	ND_2192,2202_	0.39	0.29	ND_1749,1773_	0.36	0.30
N_mass_-direct	WF_2053,3_	0.71	0.40	WF_2113,4_	0.68	0.42
	ND_2283,2286_	0.31	0.62	ND_2153,2154_	0.36	0.60
N_mass_-indirect	WF_2273,4_, WF_2060,3_	0.71	0.40	WF_1578,4_, WF_2181,4_	0.82	0.32
	ND_2236,2247_, ND_2192,2202_	0.31	0.62	ND_1716,1727_, ND_1749,1773_	0.33	0.61

The *R*^2^ and RMSE values were obtained using a 10-fold cross-validation

Note the units of RMSE were g/m^2^ for LMA and N_area_ and % for N_mass_

In addition to the 10-fold cross validation, the LOO validation accuracies are listed in Table 4. Generally, the estimation accuracy from the LOO process was lower. However, both wavelet features and ND indices derived from the WR spectra still produced higher *R*^2^ for LMA, N_area_ and the ratio of N_area_ to LMA (N_mass_ in an indirect way) than those from the MR spectra. As for N_mass_ estimation, the indirect way with wavelet features (*R*^2^ = 0.79 and RMSE = 0.34%) still worked better than the direct way (*R*^2^ = 0.61 and RMSE = 0.46%).

**Table 4:** Assessment of LMA, N_area_ and N_mass_ estimations generated with the direct ways and the indirect way

Dependent variables	Explanatory variables	MR spectra		Explanatory variables	WR spectra	
		*R* ^2^	RMSE		*R* ^2^	RMSE
LMA	WF_2273,4_	0.60	5.09	WF_1578,4_	0.63	4.88
	ND_2236,2247_	0.46	5.93	ND_1716,1727_	0.50	5.71
N_area_	WF_2060,3_	0.56	0.25	WF_2181,4_	0.72	0.19
	ND_2192,2202_	0.25	0.32	ND_1749,1773_	0.32	0.31
N_mass_-direct	WF_2053,3_	0.65	0.44	WF_2113,4_	0.64	0.44
	ND_2283,2286_	0.29	0.63	ND_2153,2154_	0.14	0.69
N_mass_-indirect	WF_2273,4_, WF_2060,3_	0.61	0.46	WF_1578,4_, WF_2181,4_	0.79	0.34
	ND_2236,2247_, ND_2192,2202_	0.01	0.74	ND_1716,1727_, ND_1749,1773_	0.26	0.64

The *R*^2^ and RMSE values were obtained using a leave-one-out validation

Not the units of RMSE were g/m^2^ for LMA and N_area_ and % for N_mass_

## Discussion

### Difference in sensitive spectral features derived from MR and WR spectra

When comparing the spectral features specifically sensitive to LMA, N_area_ and N_mass_, we found that the difference in wavelet feature was less significant between MR and WR spectra than that in ND index. The highlighted feature regions for ND indices were generally more dispersed across the scalograms, which complicated the selection of the best ND indices and was also seen in the studies by le Maire et al. [51] and Wang et al. [53]. Two dispersed index regions with even similar *R*^2^ values would lead to substantially different wavelength combinations, not to mention many of them as highlighted in Figs. 4, 7 and 9. The sensitive wavelet features were mostly concentrated in no more than two feature regions, from which it would be easier to select the best wavelet features in less than three scales and wavelength ranges. The mismatch of the wavelengths selected by ND indices with the absorption centers suggested the weakness of ND indices and the strength of wavelet features in capturing the changes in nitrogen absorption features. This might be attributed to the different calculation principles for ND indices [54] and wavelet features [33]. The former was determined by the reflectance values at two wavelengths and was sensitive to the amplitude of reflectance spectra, but the latter mainly captured the shape of a spectral segment [35]. Before and after water removal, the amplitude of spectra changed but the shapes of absorption features retained and became even more obvious. Therefore, common sensitive wavelet features can be observed between MR and WR spectra, but not for ND indices.

### Necessity of removing the effect of water absorption with CWA

The absorption of leaf water is strong in the SWIR region [19, 42, 48], hence the subtle absorption features of such dry matter constituents as protein and nitrogen are masked in the reflectance spectra of fresh leaves (Fig. 1). Many studies have shown that this masking effect reduces the estimation accuracy of these chemical constituents [14, 15, 55]. This is supported by our findings that the estimations of LMA and N_area_ were significantly improved through the wavelet-based method after the effect of water absorption had been removed. Particularly, Kokaly and Clark [11] modeled the estimation accuracy of leaf chemistry under eight levels of water content in percentage of fresh weight and found that the accuracy decreased rapidly when leaf water content exceeded 30%. To date, the effect of water absorption on ND indices and wavelet features is still poorly understood. Here we present an exploration of their performance for N_area_ estimation under different water content levels in EWT. To avoid the confounding factors of leaf surface property and internal structure, the WHEAT dataset with a wider range of EWT was selected to generate three subsets with EWT levels of 130–140 g/m^2^ (W1), 150–160 g/m^2^ (W2), and 170–180 g/m^2^ (W3).

**Fig. 12:**
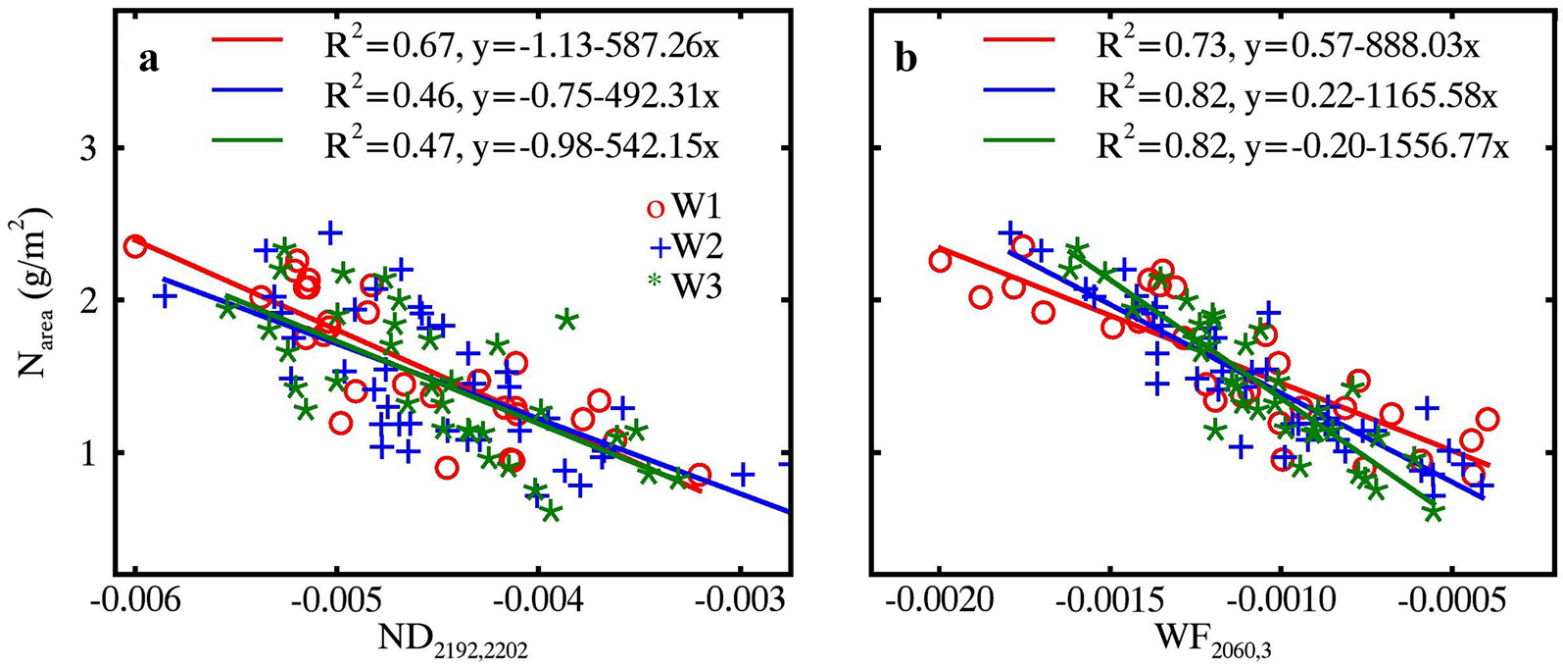
Relationships of N_area_ with **a** ND_2192,2202_ and **b** WF_2060,3_ derived from the MR spectra at three levels of EWT (W1: 130–140 g/m^2^, W2: 150–160 g/m^2^ and W3: 170–180 g/m^2^) in the WHEAT dataset. Red, blue and green lines represent linear fits for the data points from W1, W2 and W3, respectively. All regressions are statistically significant (*p* < 0.001)

The optimal index ND_2192,2202_ was well correlated to N_area_ only at the lowest EWT level (Fig. 12a), but WF_2060,3_ bore stronger relationships with N_area_ at every EWT level (Fig. 12b). In contrast to the close relationships of N_area_ and ND_2192,2202_ under three EWT levels, the N_area_ ~ WF_2060,3_ relationship was sensitive to EWT level. This is probably because WF_2060,3_ made use of the spectral information in the 2020–2100 nm range [33], which is the right wing of the water absorption valley centered at 1950 nm [19] and whose shape changes as a function of water content [11]. In spite of the tight N_area_ ~ WF_2060,3_ relationships, the sensitivity of WF_2060,3_ to EWT reduced the model transferability, e.g., from subset W1 to subset W3. Therefore, it was essential to remove water absorption for building a robust model that can be directly applied to datasets with different EWT levels.

### How did the water removal technique improve the estimations of N_area_ and N_mass_?

The accuracies of LMA and N_area_ estimations using wavelet features were improved when the effect of water absorption was removed with the water removal technique. Some recent studies used WR spectra through spectral indices or partial least squares regression to improve the estimations of LMA and N_mass_ [15, 28, 29, 56], but they did not provide explanations as to why those improvements were obtained. This study reported the application of CWA to the WR spectra for estimating foliar traits by removing the effect of water absorption and enhancing the absorption features of foliar traits. The better WF_2181,4_ ~ N_area_ relationships in the WR spectra than in the MR spectra (Fig. 6a, c) could be explained by the information on EWT in WF_2181,4_, which was derived from the MR spectra (Fig. 6d) but not from the WR spectra (Fig. 6f).

With regard to the water removal technique for N_mass_, the wavelet-based direct way did not improve the estimation accuracy but the indirect way exhibited a significant improvement (Table 3). This could be attributed to the improved estimations of both LMA and N_area_. Kokaly and Clark [11] suggested any remote sensing algorithm for retrieving foliar chemicals from fresh leaf spectra should remove the influence of water absorption. Wang et al. [56] used the normalized dry matter index (NDMI) from WR spectra to estimate the LMA but only obtained slight improvement over MR spectra as for the use of ND indices in this study (Fig. 10c, d). The wavelet features determined for LMA estimation by Cheng et al. [35] from a wide range of plant species performed better than NDMI, but they might still suffer from the effect of water absorption and be hard to be applied to other datasets with different water content levels. Therefore, the difficulty in LMA estimation hampered the indirect estimation of N_mass_ due to the dominant role of LMA as the denominator in Eq. (9). For the first time, this study obtained improved estimations for LMA as well as N_area_ by applying CWA to the WR spectra and eventually increased the estimation accuracy for N_mass_ in the indirect way.

The close fits of data points to the linear regression and the 1:1 lines in Figs. 5 and 11 suggested the best models worked well for the whole ranges of N_area_ and N_mass_. Although the rice and wheat plants in this experiment were deliberately treated in the field, the data were collected in natural light conditions and the models should be applicable to plants growing in the same condition and without any N treatment. To take advantage of the proposed strategy of combining CWA and water removal, it is imperative to have a dataset exhibiting weak covariance of EWT with LMA and N_area_. Generally, this applies to leaf level but may not apply to canopy level where the traits are determined as the products of leaf traits and leaf area index and high correlations often exist between EWT_canopy_ and other traits. In that case, it is difficult to find a wavelet feature insensitive to EWT_canopy_ but sensitive to another canopy trait. Therefore, the water removal procedure would have limited contribution to the improvement of N_mass_ or N_area_ estimation. To test the suitability of our strategy at canopy level, one would need to avoid crop datasets with high correlations of canopy EWT and nitrogen related traits. Alternatively, the strategy could be applied to natural plant types such as trees [15] and grasses [28, 29]. Although this research was conducted at leaf level, it represented a significant advance in the mechanistic understanding of water removal technique for removing water signals and enhancing nitrogen and dry matter signals in fresh leaves. The leaf-level analysis concentrated on chemical signals and laid the foundation of water removal application for scaling up to the canopy level. To better understand the mechanism at canopy level, the strategy should be examined in detail by considering other external factors such as canopy structure [57, 58], soil background [59, 60], solar zenith angle [61, 62], view zenith angle [7, 43], and diffuse/direct light condition [63]. Finally, the signal to noise ratio should also be accounted for in case the nitrogen-related second overtones are suppressed by the spectral noise from instruments and atmospheric interference [10].

### Physical interpretations of determined wavelet features

Low-scale wavelet features are able to capture the local shape of absorption features associated with biochemical parameters while high-scale ones characterize the broad continuum in leaf reflectance spectra [30, 33, 35]. The matching wavelet features could be found for N_area_ and LMA but not for N_mass_, which might suggest that the area-based expressions (N_area_ and LMA) were better representations of the interaction between matter and light per unit surface area [41].

**Fig. 13:**
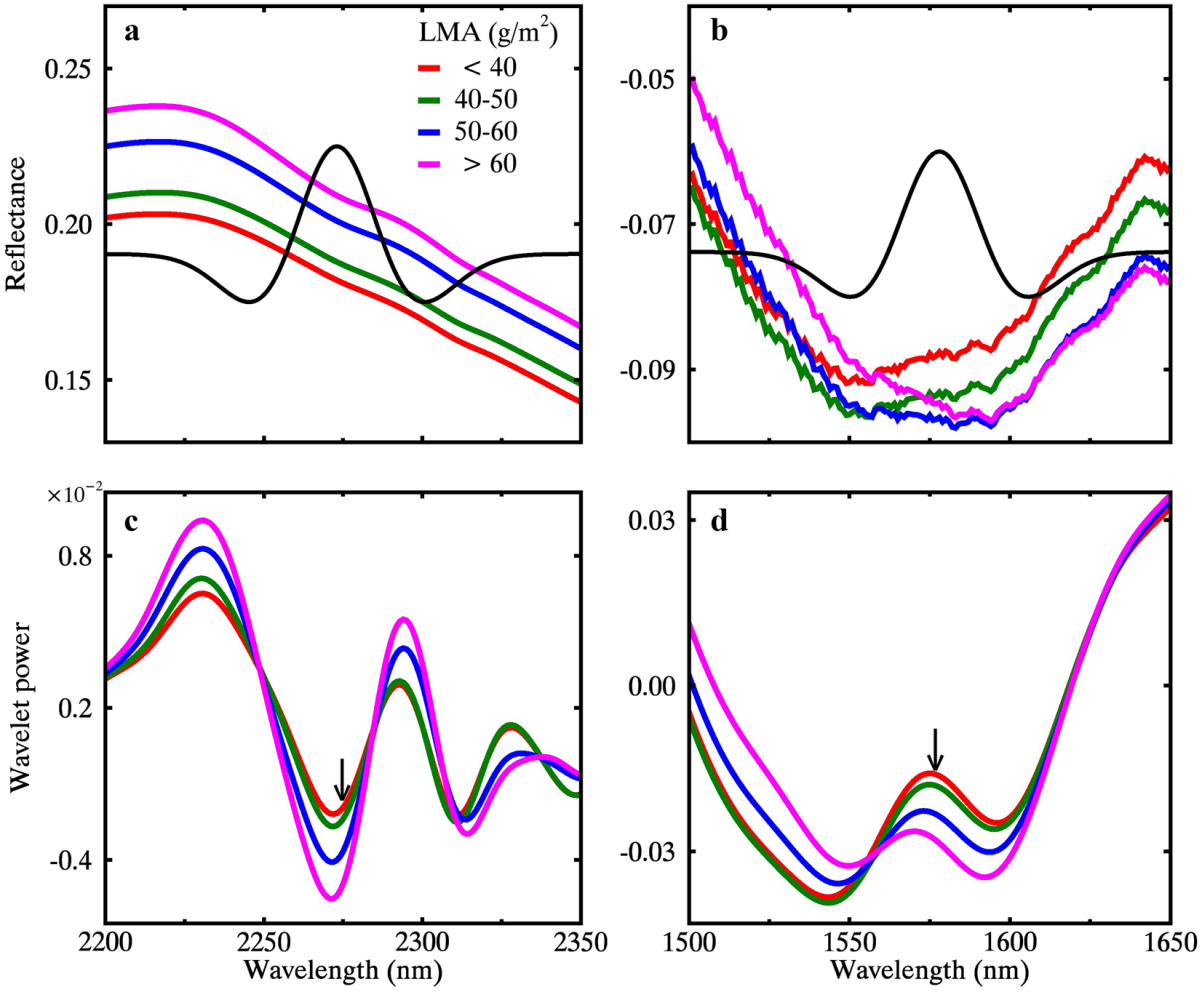
Effect of LMA on spectral variation between 2200 and 2350 nm (leaf panel) and 1500–1650 nm (right panel). **a**, **c** Represent the MR spectra and the corresponding wavelet power spectra at scale 4. **b**, **d** Represent the WR spectra and the corresponding wavelet power at scale 4. The location and width of the Gaussian-shaped wavelets for the best LMA-sensitive wavelet features WF_2273,3_ and WF_1578,4_ from MR and WR spectra are presented in (**a**) and (**b**), respectively. The black arrows in (**c**) and (**d**) point to the wavelength locations of WF_2273,3_ and WF_1578,4_, respectively

The absorption feature at 2270 nm caused by bending and stretching of chemical bonds in cellulose, sugar and starch does not obviously appear in the reflectance spectra (Figs. 1, 13a), but it is well represented in the wavelet power spectra of MR-derived WF_2273,4_ (Fig. 13c). Since LMA is equivalent to the dry matter content by definition, we could relate spectral variation to dry matter absorption from the perspective of spectroscopy of foliar chemistry as done in previous studies [35]. The amplitude of reflectance in the 2200–2350 nm range increases with LMA (Fig. 13a), which contradicts the common sense that the reflectance should decrease with dry matter absorption. This might be attributed to the leaf internal scattering as well as the confounding effect of water absorption on leaf reflectance, which makes it difficult to derive the LMA with traditional techniques, such as ND index [55, 64]. In contrast, the depth of absorption center at 2270 nm in the wavelet power spectra follows the trend of LMA variation (Fig. 13c). Moreover, the absorption feature at 1580 nm by starch and cellulose are invisible in the MR spectra (Fig. 1), but it appears clearly in the WR spectra after water removal (Fig. 13b). The change in the amplitude at WR spectra-derived WF_2273,4_ in the wavelet power spectra matches with the variation in LMA (Fig. 13d).

**Fig. 14:**
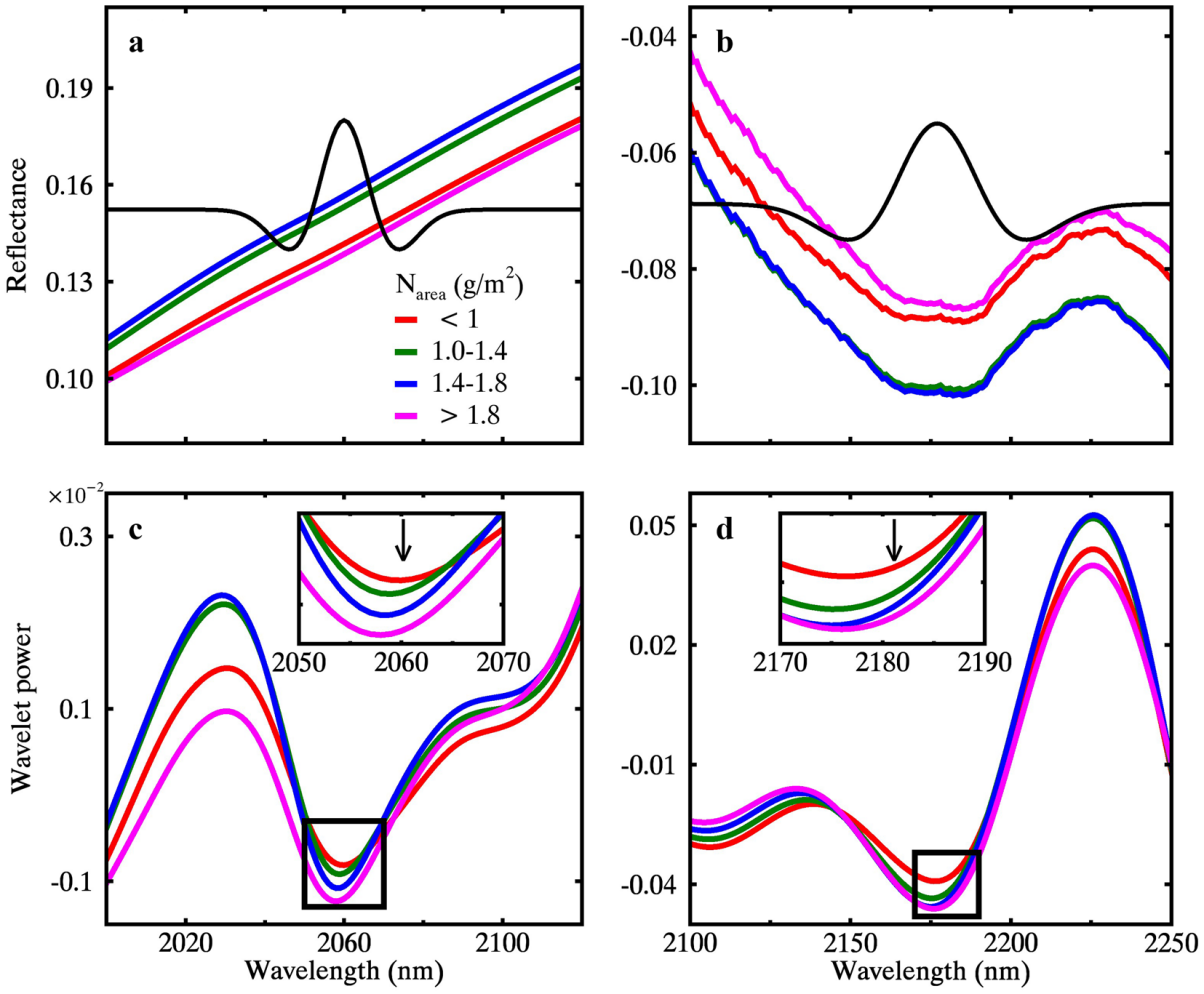
Effect of N_area_ on spectral variation between 2000 and 2120 nm (leaf panel) and 2100–2250 nm (right panel). **a**, **c** Represent the MR spectra and the corresponding wavelet power spectra at scale 3. **b**, **d** Represent the WR spectra and the corresponding wavelet power at scale 4. The location and width of the Gaussian-shaped wavelets for the best N_area_-sensitive wavelet features WF_2060,3_ and WF_2181,4_ from MR and WR spectra are presented in (**a)** and (**b**), respectively. The black arrows in (**c**) and (**d**) point to the wavelength location of the WF_2060,3_ and WF_2181,4_, respectively

Similar to dry matter, the absorption of nitrogen at 2060 nm and 2180 nm [10] could be well characterized by the optimal N_area_ sensitive wavelet features WF_2060,3_ (MR spectra) and WF_2180,4_ (WR spectra) (Fig. 14). The close matches in wavelength between the determined wavelet features and the documented absorption centers documented [10–12] convince us of the physical interpretations underlying the empirical WF-N_area_ relationships.

## Conclusions

The enhancement of absorption features of nitrogen in the SWIR region would help improve the spectroscopic estimation of leaf nitrogen content from the SWIR reflectance spectra of fresh leaves. This study evaluated the applicability of the water-removal technique for increasing the estimation accuracies of LMA, N_area_ and N_mass_ with CWA and spectral index methods. After removing water absorption, the estimation accuracies for LMA and N_area_ were significantly improved with the use of individual wavelet features but not with ND indices. The two wavelet features WF_1578,4_ and WF_2181,4_ derived from the WR spectra produced the best estimations for LMA (*R*^2^ = 0.70, RMSE = 4.38 g/m^2^) and N_area_ (*R*^2^ = 0.77, RMSE = 0.18 g/m^2^), respectively. Compared to that from the MR spectra, the estimation of N_mass_ from the WR spectra only changed marginally with individual wavelet features (MR spectra: *R*^2^ = 0.71, RMSE = 0.40%; WR spectra: *R*^2^ = 0.68, RMSE = 0.42%). However, a notable improvement was obtained when indirectly deriving N_mass_ as a ratio of N_area_ to LMA (MR spectra: *R*^2^ = 0.71, RMSE = 0.40%; WR spectra: *R*^2^ = 0.82, RMSE = 0.32%). Generally, the determined wavelet features performed remarkably better than the optimized ND indices for the estimations of all the three traits.

The results demonstrated the feasibility of improving the estimation of N_area_ and N_mass_ from the SWIR reflectance spectra by applying CWA to WR spectra. This procedure could suppress the effect of water absorption and enhance the absorption features of foliar chemicals. The characterization of nitrogen absorption variation in the SWIR region with wavelet features offers physical interpretations for the direct detection of leaf nitrogen content and complements the indirect detection of nitrogen content via the use of chlorophyll absorption bands in the red edge region. The integration of CWA and water removal provides a new insight for better understanding spectral responses to the variation in LMA and leaf nitrogen content and has the potential for applications to other crops and different vegetation types. The technique developed in this study could be useful for high throughput phenotyping of leaf nitrogen related traits and the non-destructive detection of nitrogen stress in crops.

## Abbreviations

N_area_: area-based leaf nitrogen content
N_mass_: mass-based leaf nitrogen content
LMA: leaf mass per area
WR: water-removed
CWA: continuous wavelet analysis
WF: wavelet feature
ND: normalized difference
VNIR: visible and near infrared
SWIR: shortwave infrared
*R*^2^: coefficient of determination
RMSE: root mean square error

## References

[1] Seck PA, Diagne A, Mohanty S, Wopereis MCS (2012). Crops that feed the world 7: rice. Food Secur.

[2] Shiferaw B, Smale M, Braun HJ, Duveiller E, Reynolds M, Muricho G (2013). Crops that feed the world 10. Past successes and future challenges to the role played by wheat in global food security. Food Secur.

[3] Evans JR (1989). Photosynthesis and nitrogen relationships in leaves of C_3_ plants. Oecologia.

[4] Ladha JK, Krupnik TJ, Six J, Kessel CV, Pathak H (2005). Efficiency of fertilizer nitrogen in cereal production: retrospects and prospects. Adv Agron.

[5] Gamon JA, Surfus JS (2010). Assessing leaf pigment content and activity with a reflectometer. New Phytol.

[6] Haboudane D, Miller JR, Tremblay N, Zarco-Tejada PJ, Dextraze L (2002). Integrated narrow-band vegetation indices for prediction of crop chlorophyll content for application to precision agriculture. Remote Sens Environ.

[7] He L, Song X, Feng W, Guo B-B, Zhang Y-S, Wang Y-H, Wang C-Y, Guo T-C (2016). Improved remote sensing of leaf nitrogen concentration in winter wheat using multi-angular hyperspectral data. Remote Sens Environ.

[8] Cho MA, Skidmore AK (2006). A new technique for extracting the red edge position from hyperspectral data: the linear extrapolation method. Remote Sens Environ.

[9] Huang Z, Turner BJ, Dury SJ, Wallis IR, Foley WJ (2004). Estimating foliage nitrogen concentration from HYMAP data using continuum removal analysis. Remote Sens Environ.

[10] Curran PJ (1989). Remote sensing of foliar chemistry. Remote Sens Environ.

[11] Kokaly RF, Clark RN (1999). Spectroscopic determination of leaf biochemistry using band-depth analysis of absorption features and stepwise multiple linear regression. Remote Sens Environ.

[12] Kokaly RF (2001). Investigating a physical basis for spectroscopic estimates of leaf nitrogen concentration. Remote Sens Environ.

[13] Pacheco-Labrador J, Gonzalez-Cascon R, Pilar Martin M, Riano D (2014). Understanding the optical responses of leaf nitrogen in Mediterranean Holm oak (Quercus ilex) using field spectroscopy. Int J Appl Earth Observ Geoinf.

[14] Yoder BJ, Pettigrew-Crosby RE (1995). Predicting nitrogen and chlorophyll content and concentrations from reflectance spectra (400–2500 nm) at leaf and canopy scales. Remote Sens Environ.

[15] Schlerf M, Atzberger C, Hill J, Buddenbaum H, Werner W, Schüler G (2010). Retrieval of chlorophyll and nitrogen in Norway spruce (Picea abies L. Karst.) using imaging spectroscopy. Int J Appl Earth Observ Geoinf.

[16] Cao Q, Miao Y, Feng G, Gao X, Li F, Liu B, Yue S, Cheng S, Ustin SL, Khosla R (2015). Active canopy sensing of winter wheat nitrogen status: an evaluation of two sensor systems. Comput Electron Agric.

[17] Wang Y, Wang D, Shi P, Omasa K (2014). Estimating rice chlorophyll content and leaf nitrogen concentration with a digital still color camera under natural light. Plant Methods.

[18] Schlemmer M, Gitelson A, Schepers J, Ferguson R, Peng Y, Shanahan J, Rundquist D (2013). Remote estimation of nitrogen and chlorophyll contents in maize at leaf and canopy levels. Int J Appl Earth Observ Geoinf.

[19] Jacquemoud S, Baret F (1990). PROSPECT: a model of leaf optical properties spectra. Remote Sens Environ.

[20] le Maire G, François C, Dufrêne E (2004). Towards universal broad leaf chlorophyll indices using PROSPECT simulated database and hyperspectral reflectance measurements. Remote Sens Environ.

[21] Lamb DW, Steyn-Ross M, Schaare P, Hanna MM, Silvester W, Steyn-Ross A (2002). Estimating leaf nitrogen concentration in ryegrass (*Lolium* spp.) pasture using the chlorophyll red-edge: theoretical modelling and experimental observations. Int J Remote Sens.

[22] Hansen PM, Schjoerring JK (2003). Reflectance measurement of canopy biomass and nitrogen status in wheat crops using normalized difference vegetation indices and partial least squares regression. Remote Sens Environ.

[23] Feng W, Yao X, Zhu Y, Tian YC, Cao W (2008). Monitoring leaf nitrogen status with hyperspectral reflectance in wheat. Eur J Agron.

[24] Lepine LC, Ollinger SV, Ouimette AP, Martin ME (2016). Examining spectral reflectance features related to foliar nitrogen in forests: implications for broad-scale nitrogen mapping. Remote Sens Environ.

[25] Ecarnot M, Compan F, Roumet P (2013). Assessing leaf nitrogen content and leaf mass per unit area of wheat in the field throughout plant cycle with a portable spectrometer. Field Crops Research.

[26] Hikosaka K, Terashima I (1996). Nitrogen partitioning among photosynthetic components and its consequence in sun and shade plants. Funct Ecol.

[27] Gao BC, Goetz AFH (1994). Extraction of dry leaf spectral features from reflectance spectra of green vegetation. Remote Sens Environ.

[28] Ramoelo A, Skidmore AK, Schlerf M, Mathieu R, Heitkonig IMA (2011). Water-removed spectra increase the retrieval accuracy when estimating savanna grass nitrogen and phosphorus concentrations. ISPRS J Photogramm Remote Sens.

[29] Ramoelo A, Skidmore AK, Schlerf M, Heitkonig IMA, Mathieu R, Cho MA (2013). Savanna grass nitrogen to phosphorous ratio estimation using field spectroscopy and the potential for estimation with imaging spectroscopy. Int J Appl Earth Observ Geoinf.

[30] Blackburn GA, Ferwerda JG (2008). Retrieval of chlorophyll concentration from leaf reflectance spectra using wavelet analysis. Remote Sens Environ.

[31] Li D, Cheng T, Zhou K, Zheng H, Yao X, Tian Y, Zhu Y, Cao W (2017). WREP: a wavelet-based technique for extracting the red edge position from reflectance spectra for estimating leaf and canopy chlorophyll contents of cereal crops. ISPRS J Photogramm Remote Sens.

[32] Wang HF, Huo ZG, Zhou GS, Liao QH, Feng HK, Wu L (2016). Estimating leaf SPAD values of freeze-damaged winter wheat using continuous wavelet analysis. Plant Physiol Biochem.

[33] Cheng T, Rivard B, Sánchez-Azofeifa A (2011). Spectroscopic determination of leaf water content using continuous wavelet analysis. Remote Sens Environ.

[34] Cheng T, Riaño D, Ustin SL (2014). Detecting diurnal and seasonal variation in canopy water content of nut tree orchards from airborne imaging spectroscopy data using continuous wavelet analysis. Remote Sens Environ.

[35] Cheng T, Rivard B, Sánchez-Azofeifa AG, Féret J-B, Jacquemoud S, Ustin SL (2014). Deriving leaf mass per area (LMA) from foliar reflectance across a variety of plant species using continuous wavelet analysis. ISPRS J Photogramm Remote Sens.

[36] Huang Y, Tian QJ, Wang L, Geng J, Lyu CG (2014). Estimating canopy leaf area index in the late stages of wheat growth using continuous wavelet transform. J Appl Remote Sens.

[37] Rivard B, Feng J, Gallie A, Sanchez-Azofeifa A (2008). Continuous wavelets for the improved use of spectral libraries and hyperspectral data. Remote Sens Environ.

[38] Houlès V, Guérif M, Mary B (2007). Elaboration of a nitrogen nutrition indicator for winter wheat based on leaf area index and chlorophyll content for making nitrogen recommendations. Eur J Agron.

[39] Filella I, Serrano L, Serra J, Penuelas J (1995). Evaluating wheat nitrogen status with canopy reflectance indices and discriminant analysis. Crop Sci.

[40] Shi T, Wang J, Liu H, Wu G (2015). Estimating leaf nitrogen concentration in heterogeneous crop plants from hyperspectral reflectance. Int J Remote Sens.

[41] Datt B (1998). Remote sensing of chlorophyll a, chlorophyll b, chlorophyll a + b, and total carotenoid content in Eucalyptus leaves. Remote Sens Environ.

[42] Féret J-B, François C, Asner GP, Gitelson AA, Martin RE, Bidel LPR, Ustin SL, le Maire G, Jacquemoud S (2008). PROSPECT-4 and 5: advances in the leaf optical properties model separating photosynthetic pigments. Remote Sens Environ.

[43] Jay S, Maupas F, Bendoula R, Gorretta N (2017). Retrieving LAI, chlorophyll and nitrogen contents in sugar beet crops from multi-angular optical remote sensing: comparison of vegetation indices and PROSAIL inversion for field phenotyping. Field Crops Res.

[44] Danson FM, Bowyer P (2004). Estimating live fuel moisture content from remotely sensed reflectance. Remote Sens Environ.

[45] Wang B, Chen J, Ju W, Qiu F, Zhang Q, Fang M, Chen F (2017). Limited effects of water absorption on reducing the accuracy of leaf nitrogen estimation. Remote Sens.

[46] Wright IJ, Reich PB, Westoby M, Ackerly DD, Baruch Z, Bongers F, Cavender-Bares J, Chapin T, Cornelissen JH, Diemer M (2004). The worldwide leaf economics spectrum. Nature.

[47] Li D, Cheng T, Jia M, Zhou K, Lu N, Yao X, Tian Y, Zhu Y, Cao W (2018). PROCWT: coupling PROSPECT with continuous wavelet transform to improve the retrieval of foliar chemistry from leaf bidirectional reflectance spectra. Remote Sens Environ.

[48] Ceccato P, Gobron N, Flasse S, Pinty B, Tarantola S (2002). Designing a spectral index to estimate vegetation water content from remote sensing data: part 1. Theoretical approach. Remote Sens Environ.

[49] Tsai F, Philpot W (1998). Derivative analysis of hyperspectral data. Remote Sens Environ.

[50] Inoue Y, Guerif M, Baret F, Skidmore A, Gitelson A, Schlerf M, Darvishzadeh R, Olioso A (2016). Simple and robust methods for remote sensing of canopy chlorophyll content: a comparative analysis of hyperspectral data for different types of vegetation. Plant Cell Environ.

[51] le Maire G, François C, Soudani K, Berveiller D, Pontailler J-Y, Bréda N, Genet H, Davi H, Dufrêne E (2008). Calibration and validation of hyperspectral indices for the estimation of broadleaved forest leaf chlorophyll content, leaf mass per area, leaf area index and leaf canopy biomass. Remote Sens Environ.

[52] Schlemmer MR, Francis DD, Shanahan JF, Schepers JS (2005). Remotely measuring chlorophyll content in corn leaves with differing nitrogen levels and relative water content. Agron J.

[53] Wang L, Qu JJ, Hao X, Hunt ER (2011). Estimating dry matter content from spectral reflectance for green leaves of different species. Int J Remote Sens.

[54] Rouse JW, Haas RH, Schell JA, Deering DW (1974). Monitoring vegetation systems in the great plains with Erts. NASA Spec Publ.

[55] Curran PJ, Dungan JL, Peterson DL (2001). Estimating the foliar biochemical concentration of leaves with reflectance spectrometry: testing the Kokaly and Clark methodologies. Remote Sens Environ.

[56] Wang L, ER H, Qu JJ, Hao X, Daughtry CST (2011). Estimating dry matter content of fresh leaves from the residuals between leaf and water reflectance. Remote Sens Lett.

[57] Knyazikhin Y, Schull MA, Stenberg P, Mottus M, Rautiainen M, Yang Y, Marshak A, Latorre Carmona P, Kaufmann RK, Lewis P (2013). Hyperspectral remote sensing of foliar nitrogen content. Proc Natl Acad Sci USA.

[58] Niemann KO, Quinn G, Goodenough DG, Visintini F, Loos R (2012). Addressing the effects of canopy structure on the remote sensing of foliar chemistry of a 3-dimensional, radiometrically porous surface. IEEE J Sel Top Appl Earth Observ Remote Sens.

[59] Darvishzadeh R, Skidmore A, Atzberger C, van Wieren S (2008). Estimation of vegetation LAI from hyperspectral reflectance data: effects of soil type and plant architecture. Int J Appl Earth Observ Geoinf.

[60] Yu K, Lenz-Wiedemann V, Chen X, Bareth G (2014). Estimating leaf chlorophyll of barley at different growth stages using spectral indices to reduce soil background and canopy structure effects. ISPRS J Photogramm Remote Sens.

[61] Middleton EM (1991). Solar zenith angle effects on vegetation indices in tallgrass prairie. Remote Sens Environ.

[62] Brede B, Suomalainen J, Bartholomeus H, Herold M (2015). Influence of solar zenith angle on the enhanced vegetation index of a Guyanese rainforest. Remote Sens Lett.

[63] Ishihara M, Inoue Y, Ono K, Shimizu M, Matsuura S (2015). The impact of sunlight conditions on the consistency of vegetation indices in croplands-effective usage of vegetation indices from continuous ground-based spectral measurements. Remote Sens.

[64] Grossman YL, Ustin SL, Jacquemoud S, Sanderson EW, Schmuck G, Verdebout J (1996). Critique of stepwise multiple linear regression for the extraction of leaf biochemistry information from leaf reflectance data. Remote Sens Environ.

